# Semantic Foundations for Deterministic Dataflow and Stream Processing

**DOI:** 10.1007/978-3-030-44914-8_15

**Published:** 2020-04-18

**Authors:** Konstantinos Mamouras

**Affiliations:** ETH Zurich, Zurich, Switzerland; Rice University, Houston, TX 77005 USA

**Keywords:** Data streams, Denotational semantics, Type system

## Abstract

We propose a denotational semantic framework for deterministic dataflow and stream processing that encompasses a variety of existing streaming models. Our proposal is based on the idea that data streams, stream transformations, and stream-processing programs should be classified using types. The type of a data stream is captured formally by a monoid, an algebraic structure with a distinguished binary operation and a unit. The elements of a monoid model the finite fragments of a stream, the binary operation represents the concatenation of stream fragments, and the unit is the empty fragment. Stream transformations are modeled using monotone functions on streams, which we call stream transductions. These functions can be implemented using abstract machines with a potentially infinite state space, which we call stream transducers. This abstract typed framework of stream transductions and transducers can be used to (1) verify the correctness of streaming computations, that is, that an implementation adheres to the desired behavior, (2) prove the soundness of optimizing transformations, e.g. for parallelization and distribution, and (3) inform the design of programming models and query languages for stream processing. In particular, we show that several useful combinators can be supported by the full class of stream transductions and transducers: serial composition, parallel composition, and feedback composition.

## 1 Introduction

Stream processing is the computational paradigm where the input is not presented in its entirety at the beginning of the computation, but instead it is given in an incremental fashion as a potentially unbounded sequence of elements or data items. This paradigm is appropriate in settings where data is created continually in real-time and has to be processed immediately in order to extract actionable insights and enable timely decision-making. Examples of such datasets are streams of business events in an enterprise setting [26], streams of packets that flow through computer networks [37], time-series data that is captured by sensors in healthcare applications [33], etc.

Due to the great variety of streaming applications, there are various proposals for specialized languages, compilers, and runtime systems that deal with the processing of streaming data. Relational database systems and SQL-based languages have been adapted to the streaming setting [1,2,15,16,18,19,32,37,57,91]. Recently, several systems have been developed for the distributed processing of data streams that are based on the distributed dataflow model of computation [6, 7, 70, 86, 92, 94, 108, 112, 113]. Languages for detecting complex events in distributed systems, which draw on the theory of regular expressions and finite-state automata, have also been proposed [29,40,41,50,53,88,99,111]. The synchronous dataflow formalisms [20, 24, 28, 51, 73, 107] are based on Kahn's seminal work [59], and they have been used for exposing and exploiting task-level and pipeline parallelism within streaming computations in the context of embedded systems. Several formalisms for the runtime verification of reactive systems have been proposed, many of which are based on variants of Temporal Logic and its timed/quantitative extensions [39, 43, 52, 74, 105]. Finally, there is a large collection of languages and systems for reactive programming [34,36,38,46,47,55,68,69,77,89,93,103], which focus on the development of event-driven and interactive applications such as GUIs and web programming.

The aforementioned languages and systems have been successfully used in the application domains for which they were developed. However, each one of them typically introduces a unique variant of the streaming model in terms of: (1) the form of the input and output data, (2) the class of expressible stream-processing computations, and (3) the syntax employed to describe these computations. This has resulted in an enormous proliferation of semantic models for stream processing that are difficult to compare. For this reason, we are interested in identifying a semantic unification of several existing streaming models.

This paper introduces a ***typed semantic framework*** for reasoning about languages and systems for stream processing. Three key questions are tackled:

1. How do we model *streams* and what is the form of the data that they carry?
2. How do we capture mathematically the notion of a *stream transformation*?
3. What is a general *programming model* for specifying streaming computations?

The first two questions concern the discovery of an appropriate ***denotational model*** for streaming computation. The third question concerns the design of programming and query languages, where a key requirement is that the behavior of a streaming program/query admits a precise mathematical description. Existing works have addressed these questions in the context of specific classes of applications. Here are examples of various perspectives:

– ***Transductions of strings*** [8, 100, 104, 110]: A stream is viewed as an unbounded sequence of letters, and a stream transformation is a translation from input sequences to output sequences, which is typically called string/word transduction. These translations are commonly described using finite-state transducers, a class of automata that extend acceptors with output.

– ***The streaming dataflow model of Gilles Kahn*** [59, 60]: The input and output consist of multiple independent channels that carry unbounded sequences of elements. A transformation is a function from a tuple of input sequences to a tuple of output sequences. Such transformations are specified with dataflow graphs whose nodes describe single-process computations.

– ***Relational transformations*** [71]: A stream is an unbounded multiset (bag) of tuples, and a stream transformation is a monotone operator (w.r.t. multiset containment) on multisets. This can be generalized to consider more than one input stream. An interesting subclass of these operators can be described syntactically using monotone relational algebra.

– ***Processing of time-varying relations*** [16, 17]: A stream is a time-varying finite multiset of tuples, i.e. an unbounded sequence of finite multisets of tuples. In this setting, a stream transformation processes the input in a way that preserves the notion of time: after processing $t$ input multisets (i.e., $t$ time units) the output consists of $t$ output multisets. The query language CQL [16] defines a class of such computations that involve relational and windowing operators.

– ***Transformations of continuous-time signals*** [27]: An input stream is a continuous-time signal, that is, a function from the real numbers $\mathbb{R}$ to an $n$-dimensional space $\mathbb{R}^n$. A stream transformation is a mapping from input signals to output signals that is *causal*, which means that the value of the output at time $t$ depends on the values of input signal up to (and including) time $t$. Systems of differential equations can be used to describe classes of such transformations.

We are interested here in a unifying framework that encompasses all the aforementioned concrete instances of streaming models and enables formal reasoning about the composition of streaming computations from different models. In order to achieve this we take an ***abstract algebraic approach*** that retains only the essential aspects of stream processing without any unnecessary specialization. The rest of the section outlines our proposal.

At the most fundamental level, stream processing is computation over input that is not given at the beginning in full, but rather is presented incrementally as the computation evolves. Since the input is presented piece by piece, the basic concepts that need to be captured mathematically are: (1) what is a *piece* or *fragment of the input*, and (2) how do we *extend the input*. The most general class of algebraic structures that model these notions is the class of ***monoids***, the collection of algebras that have a distinguished binary associative multiplication operation $\cdot$ and an identity element 1 for this operation. A monoid $(A, \cdot, 1)$ then constitutes a ***type of data streams***, where the elements of the monoid are all the possible *finite stream fragments*, the identity $1 \in A$ is the *empty stream* fragment, and the multiplication operation $\cdot : A \times A \to A$ models the *concatenation* of stream fragments. Using monoids, we can organize several notions of data streams using types that describe the form of the data, as well any invariants or assumptions about them. Monoids encompass the kinds of data streams that we mentioned earlier and many more: strings of letters, linear sequences of data items, tuples of sequences, multisets (bags) of data items, sets of data items, time-varying relations/multisets, (potentially disordered) timestamped sequences of data items, continuous-time signals, and so on.

Stream transformations can be classified according to the type of their input and output streams, which we call a ***transduction type***. They are modeled using *monotone functions* that map an input stream history (i.e., the fragment of the input stream that has been received from the beginning of the computation until now) to an output stream history (i.e., the fragment of the output stream produced so far). The monotonicity requirement captures the idea that a stream transformation cannot retract the output that has already been emitted. We call such functions ***stream transductions***, and we propose them as a denotational semantic model for stream processing. This model encompasses string transductions, non-diverging Kahn-computable [59] functions on streams, monotone relational transformations [71], the CQL-definable [16] transformations on time-varying relations, and transformations of continuous-time signals [27].

We also introduce an abstract model of computation for stream processing. The considered programs or abstract machines are called ***stream transducers***, and they are organized using ***transducer types*** that specify the input and output stream types. A stream transducer processes the input stream in an incremental fashion, by consuming it fragment by fragment. The consumption of an input fragment results in the emission of an output fragment. Our algebraic setting brings in an unavoidable complication compared to the classical theory of word transducers: not all stream transducers describe a stream transduction. This phenomenon has to do with the generalization of the input and output data streams from sequences of atomic data items to elements of arbitrary monoids. A stream transducer has to respect its input/output type, which means that the way in which the input stream is fragmented into pieces and fed to the transducer does not affect the cumulative output. More concisely, this says that the cumulative output is independent from the fragmentation of the input. In order to formalize this notion, we say that a *factorization* of an input history $u$ is a sequence of stream fragments $u_1, u_2, \ldots, u_n$ whose concatenation is equal to the input history, i.e. $u_1 \cdot u_2 \cdots u_n = u$. Now, the desired restriction can be described as follows: for every input history $w$ and any two factorizations $u_1, \ldots, u_m$ and $v_1, \ldots, v_m$ of $w$, the cumulative output that the transducer emits when consuming the fragments $u_1, \ldots, u_m$ in sequence is equal to the cumulative output when consuming the fragments $v_1, \ldots, v_n$. Fortunately, this complex property can be distilled into an equivalent property on the structure of the stream transducer that we call ***coherence property***. Every stream transducer that is coherent has a well-defined semantics or *denotation* in terms of a stream transduction.

We have already outlined the basics of our general framework for streaming computation, which includes: (1) a classification of streams using monoids as types, (2) a denotational semantic model that employs monotone functions from input histories to output histories, and (3) a programming model that generalizes transducers to compute meaninfully on elements of arbitrary monoids. This already allows us to address important questions about specific computations:

– Does a streaming program (transducer) behave as intended? This amounts to checking whether the denotation of the transducer is the desired function.
– Are two streaming programs (transducers) equivalent? This means that their denotations in terms of stream transductions are the same.

The first question is a *correctness* property. The second question is relevant for *semantics-preserving program optimization*. We will turn now to the issue of how to modularly specify complex stream transductions and transducers.

One of the most common ways to conceptually organize complex streaming computations is to view the overall computation as the composition of several processes that run independently and are connected with directed communication channels on which streams of data flow. This way of structuring computations is called the *dataflow programming model*. The simple deterministic parallel model of Karp and Miller [61] is one of the first variants of dataflow, and other notable early works on dataflow models include Dennis's parallel language of actors and links [42] and Kahn's networks [59] of computing stations and communication lines. We investigate three key ***dataflow combinators*** for composing stream transductions (i.e., semantic-level) and stream transducers (i.e., program-level): ***serial*** composition, ***parallel*** composition, and ***feedback*** composition. Serial composition is useful for describing pipelines of processing stages, where the output of one stage is streamed as input into the next stage. Parallel composition describes the independent and concurrent computation of two or more components. Feedback composition supports computations whose current output depends on previously produced outputs. We show that our framework supports all these combinators, which facilitate the modular description of complex computations and expose pipeline and task-based parallelism.

***Outline of paper.*** In Sect. 2 we introduce the idea that data streams can be classified using monoids as their types, and in Sect. 3 we propose the semantic model of stream transductions. Sect. 4 is devoted to the description of an abstract model of streaming computation, called stream transducer, and the main properties that it satisfies. In Sect. 5 we show that our abstract model is closed under a fundamental set of dataflow combinators: serial, parallel, and feedback composition. In Sect. 6 we prove the soundness of a streaming optimizing transformation using denotational arguments and algebraic rewriting. Sect. 7 contains related work, and Sect. 8 concludes with a brief summary of our proposal.

## 2 Monoids as Types for Streams

Data streams are typically viewed as unbounded linear sequences of data items, where a data item can be thought of as a small indivisible piece of data. This viewpoint is sufficient for describing many useful semantic and programming models, but it is too concrete and unnecessarily restricts the notion of a data stream. In order to see this, consider a computation where the specific order in which the data items arrive is not relevant. Counting is a trivial example of such a computation, and it can be described operationally as follows: every time a new data item arrives, the counting stream algorithm emits the total number of items that have been seen so far. This can be described mathematically by the function $\beta$, given by $\beta(\langle x_1, x_2, \ldots, x_n \rangle) = \langle 1, 2, \ldots, n \rangle$, where $\langle x_1, x_2, \ldots, x_n \rangle$ is the input and $\langle 1, 2, \ldots, n \rangle$ is the cumulative output of the computation. For this computation, the input can be meaningfully viewed as a *multiset* (or bag) instead of a sequence, since the ordering of the data items is irrelevant. This means that multisets can also be viewed as data streams, and in some cases this viewpoint is preferable to the traditional one of "streams = sequences".

The example of the previous paragraph raises an obvious question: What class of mathematical objects can meaningfully serve as data streams? Linear sequences and multisets should certainly be included, but it would be desirable to generalize the notion of streams as much as possible. Recent works explore the idea of generalizing streams to encompass a large class of *partial orders* [13,85], but we will see later that this approach excludes many useful instances. Stream processing is the computational paradigm where the input is not presented in full at the beginning of the computation, but instead it is given in an incremental fashion or *piece by piece*. For this reason, there are just three notions that need to be modeled mathematically: (1) a ***fragment*** or piece of a data stream, (2) the ***extension*** of data with an additional fragment of data, and (3) the ***empty*** data stream, i.e. the data seen at the very beginning of the computation. This leads us to consider a *kind* or *type of a data stream* as an algebraic structure that satisfies the following: (1) its elements model data stream fragments, (2) it has a distinguished associative operation $\cdot$ for the concatenation of stream fragments, and (3) it has a distinguished element 1 that represents the empty fragment so that 1 is a unit for concatenation. The class of monoids is the largest class of algebraic structures that fulfill these requirements.

More formally, a ***monoid*** is an algebraic structure $(A, \cdot, 1)$, where $\cdot : A \times A \to A$ is a binary operation called *multiplication* and $1 \in A$ is a constant called *unit*, that satisfies the following two axioms: (I) $(x \cdot y) \cdot z = x \cdot (y \cdot z)$ for all $x, y, z \in A$, and (II) $1 \cdot x = x \cdot 1 = x$ for all $x \in A$. The first axiom says that $\cdot$ is associative, and the second axiom says that 1 is a left and right identity for the $\cdot$ operation. For brevity, we will sometimes write $xy$ to denote $x \cdot y$.

Suppose that $A$ is a monoid. We write $A^*$ for the set of all finite sequences of elements of $A$ and $\varepsilon$ for the empty sequence. The *finite multiplication* function $\pi : A^* \to A$ is given by $\pi(\varepsilon) = 1$ and $\pi(\bar{x} \cdot \langle y \rangle) = \pi(\bar{x}) \cdot y$ for $\bar{x} \in A^*$ and $y \in A$. For sequences $\bar{x}, \bar{y} \in A^*$, it holds that $\pi(\bar{x} \cdot \bar{y}) = \pi(\bar{x}) \cdot \pi(\bar{y})$. So, $\pi$ generalizes the binary multiplication $\cdot$ to a finite but arbitrary number of arguments.

Let $(A, \cdot_A, 1_A)$ and $(B, \cdot_B, 1_B)$ be monoids. Their *product* is the monoid $(A \times B, \cdot, 1)$, where the multiplication operation is given by $(x, y) \cdot (x', y') = (x \cdot_A x', y \cdot_B y')$ for $x, x' \in A$ and $y, y' \in B$, and the identity is $1 = (1_A, 1_B)$.

A ***monoid homomorphism*** from a monoid $(A, \cdot, 1)$ to a monoid $(B, \cdot, 1)$ is a function $h : A \to B$ that commutes with the monoid operations, that is, $h(1) = 1$ and $h(x \cdot y) = h(x) \cdot h(y)$ for all $x, y \in A$.

As we discussed earlier, we can think of a monoid as a ***type of data streams***. The elements of the monoid represent *finite stream fragments*. The multiplication operation $\cdot$ models the *concatenation* of stream fragments, and the unit of the monoid is the *empty stream fragment*.

For a monoid $(A, \cdot, 1)$ we define the binary relation $\preccurlyeq$ as follows: for all $x, y \in A$, we put $x \preccurlyeq y$ if and only if $xz = y$ for some $z \in A$. Since the relation $\preccurlyeq$ is reflexive and transitive, we call it the ***prefix preorder*** for the monoid $A$. The unit 1 is a minimal element w.r.t. the $\preccurlyeq$ relation: $1 \cdot x = x$ and hence $1 \preccurlyeq x$ for every $x \in A$. Define the function $\mathsf{prefix} : A \times A \to \mathcal{P}(A)$ as follows: $\mathsf{prefix}(x, y) = \{z \in A \mid xz = y\}$ for all $x, y \in A$. This implies that $x \preccurlyeq y$ iff

$\mathsf{prefix}(x, y) \neq \emptyset$. In other words, $\mathsf{prefix}(x, y)$ is the set of all witnesses for $x \preccurlyeq y$. A partial function $\partial : A \times A \rightharpoonup A$ is said to be a *prefix witness function* (or simply a *witness function*) for the monoid $A$ if its domain is equal to $\preccurlyeq$ and it satisfies: $\partial(x, y) \in \mathsf{prefix}(x, y)$ for every $x, y \in A$ with $x \preccurlyeq y$. We can express this equivalently by requiring that the type of the function $\partial$ is $\prod_{(x,y)\in\preccurlyeq} \mathsf{prefix}(x, y)$.

We say that a monoid $A$ satisfies the *left cancellation* property if $xy = xz$ implies $y = z$ for all $x, y, z \in A$. In this case we say that $A$ is *left-cancellative.* If $A$ is left-cancellative, then it has a unique prefix witness function, because $x \preccurlyeq y$ implies that there is a unique $z$ with $xz = y$.

**Example 1 (Finite Sequences).** Consider the algebra $(\mathsf{FSeq}(A), \cdot, \varepsilon)$, where $\mathsf{FSeq}(A)$ is the set $A^*$ of all finite words (strings) over a set $A$, $\cdot$ is word concatenation, and $\varepsilon$ is the empty word. This algebra is a monoid. In fact, it is the *free monoid* with generators $A$. For $u, v \in A^*$, $u \preccurlyeq v$ iff the word $u$ is a prefix of the word $v$. There is a unique prefix witness function, because for every $x, y \in A^*$ with $x \preccurlyeq y$ there is a unique $z \in A^*$ such that $xz = y$.

Let us consider now a variant of Example 1 in order to clear any misunderstandings regarding the $\preccurlyeq$ order. The set $A^*$, together with the empty sequence $\varepsilon$, and the operation $\circ$ given by $x \circ y = yx$ is a monoid. For the monoid $(A^*, \varepsilon, \circ)$, we have that $x \preccurlyeq y$ iff $x \circ z = zx = y$ for some $z \in A^*$. So, $x \preccurlyeq y$ iff the word $x$ is a *suffix* of the word $y$.

**Example 2 (Finite Multisets).** Consider the algebra $(\mathsf{FBag}(A), \cup, \emptyset)$, where $\mathsf{FBag}(A)$ is the set of all finite multisets (bags) over a set $A$, $\cup$ is multiset union, and $\emptyset$ is the empty multiset. This algebra is a monoid. In fact, it is the *free commutative monoid* with generators $A$. It is also left cancellative. For $x, y \in \mathsf{FBag}(A)$, $x \preccurlyeq y$ iff $x$ is contained in $y$. So, we also use the notation $\subseteq$ instead of $\preccurlyeq$. There is a unique prefix witness function, because for every $x, y \in \mathsf{FBag}(A)$ with $x \subseteq y$ there is a unique $z \in \mathsf{FBag}(A)$ such that $xz = y$.

**Example 3 (Finite Sets).** Let $A$ be a set. Consider the algebra $(\mathsf{FSet}(A), \cup, \emptyset)$, where $\mathsf{FSet}(A)$ is the set of all finite subsets of $A$, $\cup$ is set union, and $\emptyset$ is the empty set. This algebra is a monoid. In fact, it is the *free commutative idempotent monoid* with generators $A$. For $x, y \in \mathsf{FBag}(A)$, $x \preccurlyeq y$ iff $x$ is contained in $y$. So, we also use the notation $\subseteq$ instead of $\preccurlyeq$.

For $x \subseteq y$, define $\partial(x, y) = y \setminus x$, where $\setminus$ is the set difference operation. Since $x \cup (y \setminus x) = y$ for $x \subseteq y$, $\partial$ is a prefix witness function. We also define $\tau(x, y) = y$ for $x \subseteq y$. Since $x \cup y = y$ for $x \subseteq y$, $\tau$ is a prefix witness function. So, $\mathsf{FSet}(A)$ has several distinct prefix witness functions.

**Example 4 (Finite Maps).** Let $K$ be a set of keys, and $V$ be a set of values. Consider the algebra $(\mathsf{FMap}(K, V), \cdot, \emptyset)$, where $\mathsf{FMap}(K, V)$ is the set of all partial maps $K \rightharpoonup V$ with a finite domain, $\emptyset$ is the partial map with empty domain, and $\cdot$ is defined as follows:

$$(f \cdot g)(k) = \begin{cases} g(k), & \text{if } g(k) \text{ is defined} \\ f(k), & \text{if } g(k) \text{ is undefined and } f(k) \text{ is defined} \\ \text{undefined}, & \text{otherwise} \end{cases}$$

for every $f, g \in \mathsf{FMap}(K, V)$ and $k \in K$. We leave it to the reader to check that $\emptyset \cdot f = f \cdot \emptyset = f$ and $(f \cdot g) \cdot h = f \cdot (g \cdot h)$ for all $f, g, h \in \mathsf{FMap}(K, V)$. So, the algebra $\mathsf{FMap}(K, V)$ is a monoid.

Let $f, g \in \mathsf{FMap}(A)$. We write $\mathrm{dom}(f) = \{k \in K \mid f(k) \text{ is defined}\}$ for the domain of $f$. It holds that $\mathrm{dom}(f \cdot g) = \mathrm{dom}(f) \cup \mathrm{dom}(g)$. Using this property, we see that $f \preccurlyeq g$ iff $\mathrm{dom}(f) \subseteq \mathrm{dom}(g)$.

Let $f, g \in \mathsf{FMap}(K, V)$ with $f \preccurlyeq g$. Define $\partial(f, g) = g$. Since $\mathrm{dom}(f) \subseteq \mathrm{dom}(g)$, we have that $f \cdot \partial(f, g) = g$. It follows that $\partial$ is a prefix witness function. Define $g \setminus f \in \mathsf{FMap}(K, V)$ as follows:

$$(g \setminus f)(k) = \begin{cases} g(k), & \text{if } g(k) \text{ is defined and } f(k) \text{ is undefined} \\ g(k), & \text{if } g(k), f(k) \text{ are defined and } g(k) \neq f(k) \\ \text{undefined}, & \text{otherwise} \end{cases}$$

for every $k \in K$. From $f \preccurlyeq g$ we get $f \cdot (g \setminus f) = g$. So, $\setminus$ is a prefix witness function. This means that $\mathsf{FMap}(K, V)$ has several distinct prefix witness functions.

**Example 5 (Bounded-Domain Continuous-Time Signals).** Let $A$ be an arbitrary set, and $\mathbb{R}$ be the set of real numbers. A bounded-domain continuous-time signal with values in $A$ is a function $f : [0, u) \to A$ where $u \geq 0$ is a real number and $[u, v) = \{t \in \mathbb{R} \mid u \leq t < v\}$. We define the *concatenation* operation $\cdot$ for such signals as follows:

$$\frac{f : [0, u) \to A \qquad g : [0, v) \to A}{f \cdot g : [0, u + v) \to A} \qquad (f \cdot g)(t) = \begin{cases} f(t), & \text{if } t \in [0, u) \\ g(t - u), & \text{if } t \in [u, u + v) \end{cases}$$

We write $\mathsf{BSig}(A)$ for the set of all these bounded-domain continuous-time signals. The *unit* signal is the unique function of type $[0, 0) \to A$, whose domain of definition is empty. Observe that $\mathsf{BSig}(A)$ is a monoid. For signals $f : [0, u) \to A$ and $g : [0, v) \to A$, it holds that $f \preccurlyeq g$ iff $u \leq v$ and $f(t) = g(t)$ for every $t \in [0, u)$. There is a unique prefix witness function, because for every $f, g \in \mathsf{BSig}(A)$ with $f \preccurlyeq g$ there is a unique $h \in \mathsf{BSig}(A)$ such that $f \cdot h = g$.

**Example 6 (Timed Finite Sequences).** We write $\mathbb{N}$ to denote the set of natural numbers (non-negative integers). A *timed sequence* over $A$ is an alternating sequence $s_0 a_1 s_1 a_2 \ldots a_n s_n$, where $s_i \in \mathbb{N}$ and $a_i \in A$ for every $i$. The occurrences $s_0, s_1, \ldots$ are called *time punctuations* and indicate the passage of time. So, the set of all timed sequences over $A$ is equal to $\mathsf{TFSeq}(A) = \mathbb{N} \cdot (A \cdot \mathbb{N})^*$. We define the *fusion product* $\diamond$ of timed sequences as follows: $s_0 a_1 s_1 \ldots a_m s_m \diamond t_0 b_1 t_1 \ldots b_n t_n = s_0 a_1 s_1 \ldots a_m (s_m + t_0) b_1 t_1 \ldots b_n t_n$. The *unit* timed sequence is the singleton sequence $0$. The algebra $(\mathsf{TFSeq}(A), \diamond, 0)$ is easily shown to be a monoid. There is a unique prefix witness function, because for all $x, y \in \mathsf{TFSeq}(A)$ with $x \preccurlyeq y$ there is a unique $z \in \mathsf{TFSeq}(A)$ s.t. $x \diamond z = y$.

**Example 7 (Finite Time-Varying Multisets).** A *finite time-varying multiset* over $A$ is a partial function $f : \mathbb{N} \rightharpoonup \mathsf{FBag}(A)$ whose domain is equal to

$[0..n] = \{0, \dots, n\}$ for some integer $n \geq 0$. We also use the notation $f : [0..n] \to \mathsf{FBag}(A)$ to convey this information regarding the domain of $f$. We define the *concatenation* operation $\cdot$ for finite time-varying multisets as follows:

$$\frac{f : [0..m] \to \mathsf{FBag}(A) \quad g : [0..n] \to \mathsf{FBag}(A)}{f \cdot g : [0..m+n] \to \mathsf{FBag}(A)} \qquad (f \cdot g)(t) = \begin{cases} f(t), & \text{if } t \in [0..m-1] \\ f(t) \cup g(0), & \text{if } t = m \\ g(t-m), & \text{if } t \in [m+1..n] \end{cases}$$

We write $\mathsf{TFBag}(A)$ to denote the set of all finite time-varying multisets over $A$. The *unit* time-varying multiset $\mathsf{Id} : [0..0] \to \mathsf{FBag}(A)$ is given by $\mathsf{Id}(0) = \emptyset$. It is easy to see that $f \cdot \mathsf{Id} = f$ and that $\mathsf{Id} \cdot f = f$ for every $f : [0..n] \to \mathsf{FBag}(A)$. We leave it to the reader to also verify that $(f \cdot g) \cdot h = f \cdot (g \cdot h)$ for finite time-varying multisets $f$, $g$ and $h$. So, the set $\mathsf{TFBag}(A)$ together with $\cdot$ and $\mathsf{Id}$ is a monoid. It is not difficult to show that it is left-cancellative.

Let us consider now the prefix preorder $\preccurlyeq$ on finite time-varying multisets. For $f : [0..m] \to \mathsf{FBag}(A)$ and $g : [0..n] \to \mathsf{FBag}(A)$, it holds that $f \preccurlyeq g$ iff $m \leq n$ and $f(t) = g(t)$ for every $t \in [0..m]$.

The examples above highlight the variety of mathematical objects that can be meaningfully viewed as streams. These streams can be organized elegantly using the structure of monoids. The sequences of Example 1, the multisets of Example 2, and the finite time-varying multisets of Example 7 can be described equivalently in terms of the partial orders of [13, 85], which have also been suggested as an approach to unify notions of streams. Using partial orders it is also possible to model the timed finite sequences of Example 6, but only with a non-succinct encoding: every time punctuation $t \in \mathbb{N}$ is encoded with a sequence $11 \dots 1$ of $t$ punctuations, one for each time unit. Partial orders cannot encode the sets of Example 3, the maps of Example 4, or the signals of Example 5. Informally, the reason for this is that partial orders can only encode *commutation equations*, which are insufficient for objects such as sets and maps.

## 3 Stream Transductions

In this section we will introduce *stream transductions* as semantic denotational models of stream transformations. At any given point in a streaming computation, we have seen an *input history* (the part of the stream from the beginning of the computation until now) and we have produced an *output history* (the cumulative output that has been emitted from the beginning until now). As a first approximation, a streaming computation can be described mathematically by a function $\beta : A \to B$, where $A$ and $B$ are monoids that describe the input and output type respectively, which maps an input history $x \in A$ to an output history $\beta(x) \in B$. The function $\beta$ has to be *monotone* because the output is cumulative, which means that it can only be extended with more output items as the computation proceeds. An equivalent way to understand the monotonicity property is that it captures the idea that any output that has already been emitted cannot be retracted. Since $\beta$ takes an entire input history as its argument, it can describe stateful computations, where the output that is emitted at every step potentially depends on the entire input history.

**Definition 8 (Stream Transduction & Incremental Form).** Let $A$ and $B$ be monoids. A function $\beta : A \to B$ is said to be *monotone* (with respect to the prefix preorder) if $x \preccurlyeq y$ implies $\beta(x) \preccurlyeq \beta(y)$ for all $x, y \in A$. For a monotone $\beta : A \to B$, we say that the partial function $\mu$ is a *monotonicity witness function* if it maps elements $x, y \in A$ and $z \in \mathsf{prefix}(x, y)$ witnessing that $x \preccurlyeq y$ to a witness $\mu(x, y, z) \in \mathsf{prefix}(\beta(x), \beta(y))$ for $\beta(x) \preccurlyeq \beta(y)$. That is, we require that the type of $\mu$ is $\prod_{x,y \in A} \mathsf{prefix}(x, y) \to \mathsf{prefix}(\beta(x), \beta(y))$. So, the defining property of $\mu$ is that for all $x, y, z \in A$ with $xz = y$ it holds that $\beta(x) \cdot \mu(x, y, z) = \beta(y)$. For brevity, we will sometimes write $\mu(x, z)$ to denote $\mu(x, xz, z)$. The defining property of $\mu$ is then written as $\beta(x) \cdot \mu(x, z) = \beta(xz)$ for all $x, z \in A$.

A ***stream transduction*** from $A$ to $B$ is a function $\beta : A \to B$ that is monotone with respect to the prefix preorder, together with a monotonicity witness function $\mu : \prod_{x,y \in A} \mathsf{prefix}(x, y) \to \mathsf{prefix}(\beta(x), \beta(y))$. We write $\mathsf{STrans}(A, B)$ to denote the set of all stream transductions from $A$ to $B$.

The ***incremental form*** of a stream transduction $\langle \beta, \mu \rangle \in \mathsf{STrans}(A, B)$ is a function $\mathsf{F}(\beta, \mu) : A^* \to B^*$, which is defined inductively by $\mathsf{F}(\beta, \mu)(\varepsilon) = \langle \beta(1) \rangle$ and $\mathsf{F}(\beta, \mu)(\langle x_1, \ldots, x_n, x_{n+1} \rangle) = \mathsf{F}(\beta, \mu)(\langle x_1, \ldots, x_n \rangle) \cdot \langle \mu(x_1 \cdots x_n, x_{n+1}) \rangle$ for every sequence $\langle x_1, \ldots, x_{n+1} \rangle \in A^*$.

Consider the stream transduction $\langle \beta, \mu \rangle : \mathsf{STrans}(A, B)$ and the input fragments $x, y \in A$. Notice that $\mu(x, y)$ gives the *output increment* that the streaming computation generates when the input history $x$ is extended into $xy$. For an arbitrary output monoid $B$, the output increment $\mu(x, y)$ is generally not uniquely determined by $\beta(x)$ and $\beta(xy)$. This means that the monotonicity witness function $\mu$ generally provides some additional information about the streaming computation that cannot be obtained purely from $\beta$. However, if the output monoid $B$ is left-cancellative then there is a unique function $\mu$ that witnesses the monotonicity of $\beta$.

Suppose that $\langle \beta, \mu \rangle : \mathsf{STrans}(A, B)$ is a stream transduction. The incremental form $\mathsf{F}(\beta, \mu)$ of the transduction $\langle \beta, \mu \rangle$ describes the stream transformation in explicit input/output increments. For example, $\mathsf{F}(\beta, \mu)(\langle x_1 \rangle) = \langle \beta(1), \mu(1, x_1) \rangle$ and $\mathsf{F}(\beta, \mu)(\langle x_1, x_2 \rangle) = \langle \beta(1), \mu(1, x_1), \mu(x_1, x_2) \rangle$. The key property of the incremental form is that $\pi(\mathsf{F}(\beta, \mu)(\bar{x})) = \beta(\pi(\bar{x}))$ for every $\bar{x} \in A^*$. For example, $\pi(\mathsf{F}(\beta, \mu)(\langle x_1, x_2, x_3 \rangle)) = \beta(1) \cdot \mu(1, x_1) \cdot \mu(x_1, x_2) \cdot \mu(x_1 x_2, x_3) = \beta(x_1) \cdot \mu(x_1, x_2) \cdot \mu(x_1 x_2, x_3) = \beta(x_1 x_2) \cdot \mu(x_1 x_2, x_3) = \beta(x_1 x_2 x_3)$.

**Example 9 (Counting).** Let $A$ be an arbitrary set. We will describe a streaming computation whose input type is the monoid $\mathsf{FBag}(A)$ and whose output type is the monoid $\mathsf{FSeq}(\mathbb{N})$. The informal operational description is as follows: there is no initial output, and every time a new data item arrives the computation emits the total number of items seen so far. The formal description is given by the stream transduction $\beta : \mathsf{FBag}(A) \to \mathsf{FSeq}(\mathbb{N})$, defined by $\beta(\emptyset) = \varepsilon$ and $\beta(x) = \langle 1, 2, \ldots, |x| \rangle$ for every non-empty $x \in \mathsf{FBag}(A)$, where $|x|$ denotes the size of the multiset $x$. It is easy to see that $\beta$ is monotone. Since $\mathsf{FSeq}(\mathbb{N})$

is left-cancellative, the monotonicity witness function is uniquely determined: $\mu(x, \emptyset) = \varepsilon$ and $\mu(x, y) = \langle |x| + 1, \ldots, |x| + |y| \rangle$ when $y \neq \emptyset$.

**Example 10 (Per-Key Aggregation).** Let $K$ be a set of keys, and $V$ be a set of values. The elements of $K \times V$ are typically called key-value pairs. Suppose that $\mathsf{op} : V \times V \to V$ is an associative and commutative operation. So, $\mathsf{op}$ can be generalized to an aggregation operation that takes non-empty finite multisets over $V$ as input. We will describe a streaming computation whose input type is the monoid $\mathsf{FBag}(K \times V)$ and whose output type is the monoid $\mathsf{FMap}(K, V)$. Informally, every time an item $(k, v)$ is processed, the output map is updated so that the $k$-indexed entry contains the aggregate (using $\mathsf{op}$) of all values seen so far for the key $k$. The formal description of this computation is given by the stream transduction $\beta : \mathsf{FBag}(K \times V) \to \mathsf{FMap}(K, V)$, defined by $\beta(x) = \{k \mapsto \mathsf{op}(x|_k) \mid k \text{ appears in } x\}$ for every multiset $x$, where $x|_k$ denotes the multiset that results from $x$ by keeping only the pairs whose key is equal to $k$. That is, the domain of $\beta(x)$ is equal to $\mathrm{dom}(\beta(x)) = \{k \in K \mid k \text{ appears in } x\}$ and $\beta(x)(k) = \mathsf{op}(x|_k)$ for every $k$ that appears in $x$. The monotonicity witness function $\mu$ is defined as follows: $\mu(x, y)$ is equal to the restriction of the map $\beta(x \cup y)$ to the set of all keys that appear in $y$.

We saw in Sect. 2 that we can form products of monoids: if $A$ and $B$ are monoids, then so is $A \times B$. Intuitively, we can think of $A \times B$ as the data stream type that involves two parallel and independent *channels*: one channel for streams of type $A$ and another channel for streams of type $B$.

**Example 11 (Merging of Multiple Input Channels).** Given a set $A$, we want to describe a transformation with two input channels of type $\mathsf{FBag}(A)$ and one output channel of type $\mathsf{FBag}(A)$. The monotone function $\beta : \mathsf{FBag}(A) \times \mathsf{FBag}(A) \to \mathsf{FBag}(A)$, given by $\beta(x, y) = x \cup y$ for multisets $x$ and $y$, describes the merging of the two input substreams. Operationally, whenever a new data item arrives (regardless of channel) it is propagated to the output channel. Since $\mathsf{FBag}(A)$ is left-cancellative, the monotonicity witness function is uniquely determined: $\mu(\langle x_1, y_1 \rangle, \langle x_2, y_2 \rangle) = (x_2 \cup y_2) \setminus (x_1 \cup y_1)$ for all $x_1, y_1, x_2, y_2 \in \mathsf{FBag}(A)$.

**Example 12 (Flatten).** Let $A$ be a monoid. The function $\beta : \mathsf{FSeq}(A) \to A$, given by $\beta(\bar{x}) = \pi(\bar{x})$ for every $\bar{x} \in \mathsf{FSeq}(A)$, describes the *flattening* of a sequence of monoid elements. The function $\beta$ is monotone, and its monotonicity witness function $\mu$ is given by $\mu(\bar{x}, \bar{y}) = \pi(\bar{y})$ for all $\bar{x}$ and $\bar{y}$. The stream transduction $\mathit{flatten}(A) = \langle \beta, \mu \rangle$ has type $\mathsf{STrans}(\mathsf{FSeq}(A), A)$.

**Example 13 (Split in Batches).** Let $\Sigma = \{a, b\}$ be an alphabet of symbols. Suppose that we want to describe the decomposition of an element of $\Sigma^*$ into batches of size exactly 3. We describe this using two functions $r_1 : \Sigma^* \to \mathsf{FSeq}(\Sigma^*)$ and $r_2 : \Sigma^* \to \Sigma^*$. Informally, $r_1$ gives the sequence of full batches of size 3, and $r_2$ gives the remaining incomplete batch. For example, $r_1(abbaabba) = \langle abb, aab \rangle$ and $r_2(abbaabba) = ba$.

This idea of splitting in batches can be generalized from the monoid $\Sigma^*$ to an arbitrary monoid $A$. We say that a ***splitter*** for $A$ is a pair $r = (r_1, r_2)$ of functions $r_1 : A \to \mathsf{FSeq}(A)$ and $r_2 : A \to A$ satisfying the following properties: (1) the equality $x = \pi(r_1(x)) \cdot r_2(x)$ says that $r_1$ and $r_2$ decompose $x \in A$, (2) $r_1(1_A) = \varepsilon$ says that the unit cannot be decomposed, (3) $r_1(x \cdot y) = r_1(x) \cdot r_1(r_2(x) \cdot y)$ and (4) $r_2(x \cdot y) = r_2(r_2(x) \cdot y)$ describe how to decompose the concatenation of two monoid elements. The first two properties imply that $r_2(1_A) = 1_A$. The third property implies that $r_1$ is monotone. Define $\mu(x, y) = r_1(r_2(x) \cdot y)$ for $x, y \in A$ and observe that $r_1(x) \cdot \mu(x, y) = r_1(xy)$. It follows that $\mathit{split}(r) = \langle r_1, \mu\rangle$ is a stream transduction of type $\mathsf{STrans}(A, \mathsf{FSeq}(A))$.

Our denotational model of a stream transformation uses a monotone function whose domain is the monoid of (finite) input histories. We emphasize that such a denotation can also describe the transformation of an ***infinite stream***. To illustrate this point in simple terms, consider a monotone function $\beta : A^* \to B^*$, where $A$ (resp., $B$) is the type of input (resp., output) items. This function extends uniquely to the $\omega$-continuous function $\beta^\infty : A^\infty \to B^\infty$, where $A^\infty = A^* \cup A^\omega$ is the set of finite and infinite sequences over $A$, as follows: $\beta^\infty(a_0 a_1 a_2 \ldots)$ is equal to the supremum of the chain $\beta(\varepsilon) \leq \beta(a_0) \leq \beta(a_0 a_1) \leq \ldots$

## 4 Model of Computation

We will present an abstract model of computation for stream processing, where the input and output data streams are elements of monoids $A$ and $B$ respectively. A streaming algorithm is described by a transducer, a kind of automaton that produces output values. We consider transducers that can have a potentially infinite state space, which we denote by $\mathsf{St}$. The computation starts at a distinguished initial state $\mathsf{init} \in \mathsf{St}$, and the initialization triggers some initial output $\mathsf{o} \in B$. The computation then proceeds by consuming the input stream incrementally, i.e. fragment by fragment. One step of the computation from a state $s \in \mathsf{St}$ involves consuming an input fragment $x \in A$, producing an output increment $\mathsf{out}(s, x) \in B$ and transitioning to the next state $\mathsf{next}(s, x) \in \mathsf{St}$.

**Definition 14 (Stream Transducer).** Let $A$, $B$ be monoids. A *stream transducer* with inputs from $A$ and outputs from $B$ is a tuple $\mathcal{G} = (\mathsf{St}, \mathsf{init}, \mathsf{o}, \mathsf{next}, \mathsf{out})$, where $\mathsf{St}$ is a nonempty set of *states*, $\mathsf{init} \in \mathsf{St}$ is the *initial state*, $\mathsf{o} \in B$ is the *initial output*, $\mathsf{next} : \mathsf{St} \times A \to \mathsf{St}$ is the *transition function*, and $\mathsf{out} : \mathsf{St} \times A \to B$ is the *output function*. We write $\mathsf{G}(A, B)$ to denote the set of all stream transducers with inputs from $A$ and outputs from $B$.

We define the *generalized transition function* $\mathsf{gnext} : \mathsf{St} \times A^* \to \mathsf{St}$ by induction: $\mathsf{gnext}(s, \varepsilon) = s$ and $\mathsf{gnext}(s, \langle x\rangle \cdot \bar{y}) = \mathsf{gnext}(\mathsf{next}(s, x), \bar{y})$ for all $s \in \mathsf{St}$, $x \in A$ and $\bar{y} \in A^*$. A state $s \in \mathsf{St}$ is said to be *reachable* in $\mathcal{G}$ if there exists a sequence $\bar{x} \in A^*$ such that $\mathsf{gnext}(\mathsf{init}, \bar{x}) = s$.

We define the *generalized output function* $\mathsf{gout} : \mathsf{St} \times A^* \to B$ by induction on the second argument: $\mathsf{gout}(s, \varepsilon) = 1$ and $\mathsf{gout}(s, \langle x\rangle \cdot \bar{y}) = \mathsf{out}(s, x) \cdot \mathsf{gout}(\mathsf{next}(s, x), \bar{y})$ for all $s \in \mathsf{St}$, $x \in A$ and $\bar{y} \in A^*$. The *extended output function* $\mathsf{eout} : \mathsf{St} \times A^* \to B^*$ is defined similarly: $\mathsf{eout}(s, \varepsilon) = \varepsilon$ and $\mathsf{eout}(x, \langle x\rangle \cdot \bar{y}) = \langle \mathsf{out}(s, x)\rangle \cdot \mathsf{eout}(\mathsf{next}(s, x), \bar{y})$ for all $s \in \mathsf{St}$, $x \in A$ and $\bar{y} \in A^*$.

**Example 15 (Transducer for Counting).** Recall the counting streaming computation that was described in Example 9. We will describe a stream transducer that implements the counting computation. The input monoid is $\mathsf{FBag}(A)$ and the output monoid is $\mathsf{FSeq}(\mathbb{N})$. The state space is $\mathsf{St} = \mathbb{N}$, because the transducer has to maintain a counter that remembers the number of data items seen so far. The initial state is $\mathsf{init} = 0$ and the initial output is $\mathsf{o} = \varepsilon$. The transition function increments the counter, i.e. $\mathsf{next}(s, x) = s + |x|$ for every $s \in \mathsf{St}$ and $x \in \mathsf{FBag}(A)$. The output function is defined by $\mathsf{out}(s, \emptyset) = \varepsilon$ and $\mathsf{out}(s, x) = \langle s + 1, \ldots, s + |x| \rangle$ for a nonempty multiset $x$. The type of this transducer is $\mathsf{G}(\mathsf{FBag}(A), \mathsf{FSeq}(\mathbb{N}))$.

**Example 16 (Transducer for Merging).** We will implement the merging computation of Example 11, where there are two input channels of type $\mathsf{FBag}(A)$ and one output channel of type $\mathsf{FBag}(A)$. The transducer does not need memory, so $\mathsf{St} = \mathsf{Unit}$, where $\mathsf{Unit} = \{\star\}$ is a singleton set. The initial state is $\mathsf{init} = \star$ and the initial output is $\mathsf{o} = \emptyset$. There is only one possibility for the transition function: $\mathsf{next}(s, \langle x, y \rangle) = \star$. The output function describes the propagation of the input increments of both input channels to the output channel: $\mathsf{out}(s, \langle x, y \rangle) = x \cup y$ for all multisets $x, y$. The type of this transducer is $\mathsf{G}(\mathsf{FBag}(A) \times \mathsf{FBag}(A), \mathsf{FBag}(A))$.

**Example 17 (Flatten).** For a monoid $A$, we define a transducer $\mathtt{Flatten}(A) = (\mathsf{St}, \mathsf{init}, \mathsf{o}, \mathsf{next}, \mathsf{out}) : \mathsf{G}(\mathsf{FSeq}(A), A)$ that implements the flattening transduction of Example 12. This computation does not require memory, so we define $\mathsf{St} = \mathsf{Unit}$ and $\mathsf{init} = \star$. The initial output is $\mathsf{o} = 1_A$, the transition function is uniquely determined by $\mathsf{next}(s, x) = \star$, and the output function is given by $\mathsf{out}(s, \langle a_1, \ldots, a_n \rangle) = a_1 \cdots a_n$.

**Example 18 (Split in Batches).** For a monoid $A$ and a splitter $r = (r_1, r_2)$ for $A$ (Example 13), we describe a transducer $\mathtt{Split}(r) = (\mathsf{St}, \mathsf{init}, \mathsf{o}, \mathsf{next}, \mathsf{out})$ that implements the transduction $\mathit{split}(r) : \mathsf{STrans}(A, \mathsf{FSeq}(A))$. We define $\mathsf{St} = A$, because the transducer needs to remember the remainder of the cumulative input that does not yet form a complete batch, and $\mathsf{init} = 1_A$. The initial output $\mathsf{o} = \varepsilon$ is the empty sequence. The transition and output functions are defined by $\mathsf{next}(s, x) = r_2(s \cdot x)$ and $\mathsf{out}(s, x) = r_1(s \cdot x)$.

Definition 14 does not capture a key requirement for streaming computations over monoids, namely that the cumulative output of a transducer $\mathcal{G}$ should be independent of the particular way in which the input history is split into the fragments that are fed to it. More precisely, suppose that $w$ is an input history that can be fragmented (factorized) in two different ways: $w = u_1 \cdot u_2 \cdots u_m$ and $w = v_1 \cdot v_2 \cdots v_n$. Then, the cumulative output of the transducer $\mathcal{G}$ when consuming the sequence of fragments (factorization) $u_1, u_2, \ldots, u_m$ should be equal to the cumulative output when consuming $v_1, v_2, \ldots, v_n$. In Definition 20 below, we formulate a set of *coherence conditions* that a transducer must adhere to in order to satisfy this "factorization independence" requirement.

**Definition 19 (Bisimulation & Bisimilarity).** Let $\mathcal{G} = (\mathsf{St}, \mathsf{init}, \mathsf{o}, \mathsf{next}, \mathsf{out})$ be a transducer with inputs from $A$ and outputs from $B$. A relation $R \subseteq \mathsf{St} \times \mathsf{St}$ is a *bisimulation* for $\mathcal{G}$ if for every $s, t \in \mathsf{St}$ and $x \in A$ we have that $(s,t) \in R$ implies $\mathsf{out}(s,x) = \mathsf{out}(t,x)$ and $(\mathsf{next}(s,x), \mathsf{next}(t,x)) \in R$. We will also use the notation $sRt$ to mean $(s,t) \in R$. We say that the states $s, t \in R$ are *bisimilar*, denoted $s \sim t$, if there exists a bisimulation $R$ for $\mathcal{G}$ such that $sRt$. The relation $\sim$ is called the *bisimilarity relation* for $\mathcal{G}$.

It is well-known that the bisimilarity relation for $\mathcal{G}$ is an equivalence relation (reflexive, symmetric, and transitive), and for all $s, t \in \mathsf{St}$ and $x \in A$ it satisfies the following ***extension property***: $s \sim t$ implies that $\mathsf{next}(s,x) \sim \mathsf{next}(t,x)$. It can then be easily seen that the bisimilarity relation is a bisimulation. In fact, it is the largest bisimulation for the transducer $\mathcal{G}$.

**Definition 20 (Coherence).** Suppose $\mathcal{G} = (\mathsf{St}, \mathsf{init}, \mathsf{o}, \mathsf{next}, \mathsf{out}) : \mathsf{G}(A, B)$ is a stream transducer. We say that $\mathcal{G}$ is *coherent* if it satisfies the following:
(N1) $\mathsf{next}(\mathsf{init}, 1) \sim \mathsf{init}$.
(N2) $\mathsf{next}(\mathsf{init}, xy) \sim \mathsf{next}(\mathsf{next}(\mathsf{init}, x), y)$ for every $x, y \in A$.
(O1) $\mathsf{o} \cdot \mathsf{out}(\mathsf{init}, 1) = \mathsf{o}$.
(O2) $\mathsf{o} \cdot \mathsf{out}(\mathsf{init}, xy) = \mathsf{o} \cdot \mathsf{out}(\mathsf{init}, x) \cdot \mathsf{out}(\mathsf{next}(\mathsf{init}, x), y)$ for every $x, y \in A$.

The coherence conditions of Definition 20 capture the idea that the transducer behaves in "essentially the same way" regardless of how the input is split into fragments. For example, the condition (N2) says that the two-step transition $\mathsf{init} \to^x s_1 \to^y s_2$ and the single-step transition $\mathsf{init} \to^{xy} t_1$ end up in states ($s_2$ and $t_1$) that will have exactly the same behavior in the subsequent computation. In other words, it does not matter whether the input $xy$ was fed to the transducer as a single fragment $xy$ or as a sequence of two fragments $\langle x, y \rangle$.

Let $(A, \cdot, 1)$ be a monoid. A *factorization* of an element $x \in A$ is a sequence $x_1, \ldots, x_n$ of elements of $A$ such that $x = x_1 \cdots x_n$. In particular, the empty sequence $\varepsilon \in A^*$ is a factorization of 1. In other words, $\bar{x} \in A^*$ is a factorization of $x \in A$ if $\pi(\bar{x}) = x$.

**Theorem 21 (Factorization Independence).** Let $\mathcal{G} = (\mathsf{St}, \mathsf{init}, \mathsf{o}, \mathsf{next}, \mathsf{out})$ be a stream transducer of type $\mathsf{G}(A, B)$. If $\mathcal{G}$ is coherent, then for every $x \in A$ and every factorization $\bar{x} \in A^*$ of $x$ we have that $\mathsf{o} \cdot \mathsf{gout}(\mathsf{init}, \bar{x}) = \mathsf{o} \cdot \mathsf{out}(\mathsf{init}, x)$.

*Proof.* For clarity, we write $\langle x_1, x_2, \ldots, x_n \rangle \in A^*$ to denote a finite sequence of elements of $A$. The following properties hold for all $s \in \mathsf{St}$, $\bar{x} \in A^*$ and $y \in A$:

$$\mathsf{gnext}(s, \bar{x} \cdot \langle y \rangle) = \mathsf{next}(\mathsf{gnext}(s, \bar{x}), y) \tag{1}$$

$$\mathsf{gout}(s, \bar{x} \cdot \langle y \rangle) = \mathsf{gout}(s, \bar{x}) \cdot \mathsf{out}(\mathsf{gnext}(s, \bar{x}), y) \tag{2}$$

$$\mathsf{eout}(s, \bar{x} \cdot \langle y \rangle) = \mathsf{eout}(s, \bar{x}) \cdot \langle \mathsf{out}(\mathsf{gnext}(s, \bar{x}), y) \rangle \tag{3}$$

Each property shown above can be proved by induction on the sequence $\bar{x}$.

Consider an arbitrary *coherent* stream transducer $\mathcal{G} = (\mathsf{St}, \mathsf{init}, \mathsf{o}, \mathsf{next}, \mathsf{out})$. We claim that $\mathcal{G}$ satisfies the following coherence property:

$$\mathsf{gnext}(\mathsf{init}, \langle x_1, \ldots, x_n \rangle) \sim \mathsf{next}(\mathsf{init}, x_1 \cdots x_n) \text{ for all } \langle x_1, \ldots, x_n \rangle \in A^*. \tag{N*}$$

The proof is by induction on the length of the sequence. For the base case, we have that $\mathsf{gnext}(\mathsf{init}, \varepsilon) = \mathsf{init}$ and $\mathsf{next}(\mathsf{init}, 1)$ are bisimilar because $\mathcal{G}$ is coherent (recall Property (N1) of Definition 20). For the induction step we have:

$$\begin{aligned} \mathsf{gnext}(\mathsf{init}, \bar{x} \cdot \langle y \rangle) &= \mathsf{next}(\mathsf{gnext}(\mathsf{init}, \bar{x}), y) && \text{[Equation (1)]} \\ &\sim \mathsf{next}(\mathsf{next}(\mathsf{init}, \pi(\bar{x})), y) && \text{[I.H., extension]} \\ &\sim \mathsf{next}(\mathsf{init}, \pi(\bar{x}) \cdot y), && \text{[coherence (N2)]} \end{aligned}$$

which is equal to $\mathsf{next}(\mathsf{init}, \pi(\bar{x} \cdot \langle y \rangle))$. This concludes the proof of the claim (N*).

The proof of the theorem proceeds by induction on $\bar{x} \in A^*$. For the base case, observe that $\mathsf{o} \cdot \mathsf{gout}(\mathsf{init}, \varepsilon) = \mathsf{o} \cdot 1 = \mathsf{o}$ is equal to $\mathsf{o} \cdot \mathsf{out}(\mathsf{init}, 1) = \mathsf{o}$ (property (O1) for $\mathcal{G}$). For the induction step, we have:

$$\begin{aligned} \mathsf{o} \cdot \mathsf{gout}(\mathsf{init}, \bar{x} \cdot \langle y \rangle) &= \mathsf{o} \cdot \mathsf{gout}(\mathsf{init}, \bar{x}) \cdot \mathsf{out}(\mathsf{gnext}(\mathsf{init}, \bar{x}), y) && \text{[Eq. (2)]} \\ &= \mathsf{o} \cdot \mathsf{out}(\mathsf{init}, \pi(\bar{x})) \cdot \mathsf{out}(\mathsf{gnext}(\mathsf{init}, \bar{x}), y) && \text{[I.H.]} \\ &= \mathsf{o} \cdot \mathsf{out}(\mathsf{init}, \pi(\bar{x})) \cdot \mathsf{out}(\mathsf{next}(\mathsf{init}, \pi(\bar{x})), y) && \text{[Prop. (N*)]} \\ &= \mathsf{o} \cdot \mathsf{out}(\mathsf{init}, \pi(\bar{x}) \cdot y) && \text{[Prop. (O2)]} \end{aligned}$$

which is equal to $\mathsf{o} \cdot \mathsf{out}(\mathsf{init}, \pi(\bar{x} \cdot \langle y \rangle))$. □

Theorem 21 says that the condition of coherence guarantees a basic correctness property for stream transducers: the output that they produce does not depend on the specific way in which the input was partitioned into fragments.

For a transducer $\mathcal{G} = (\mathsf{St}, \mathsf{init}, \mathsf{o}, \mathsf{next}, \mathsf{out})$ we define the function $[\![G]\!] : A^* \to B^*$ as follows: $[\![G]\!](\bar{x}) = \langle \mathsf{o} \rangle \cdot \mathsf{eout}(\mathsf{init}, \bar{x})$ for every $\bar{x} \in A^*$. We call $[\![\mathcal{G}]\!]$ the ***interpretation*** or ***denotation*** of $\mathcal{G}$. The definition of $[\![\mathcal{G}]\!]$ implies that $[\![\mathcal{G}]\!](\varepsilon) = \langle \mathsf{o} \rangle$ and the following holds for every $\bar{x} \in A^*$ and $y \in A$:

$$[\![\mathcal{G}]\!](\bar{x} \cdot \langle y \rangle) = [\![\mathcal{G}]\!](\bar{x}) \cdot \langle \mathsf{out}(\mathsf{gnext}(\mathsf{init}, \bar{x}), y) \rangle \tag{4}$$

When $\mathcal{G}$ is coherent, Theorem 21 says that the denotation gives the same cumulative output for any two factorizations of the input. We say that the transducers $\mathcal{G}_1$ and $\mathcal{G}_2$ are ***equivalent*** if their denotations are equal, i.e. $[\![\mathcal{G}_1]\!] = [\![\mathcal{G}_2]\!]$.

**Definition 22 (The Implementation Relation).** Let $A, B$ be monoids, $\mathcal{G} : \mathsf{G}(A, B)$ be a stream transducer, and $\langle \beta, \mu \rangle : \mathsf{STrans}(A, B)$ be a stream transduction. We say that $\mathcal{G}$ *implements* $\langle \beta, \mu \rangle$ if $[\![\mathcal{G}]\!](\bar{x}) = \mathsf{F}(\beta, \mu)(\bar{x})$ for every $\bar{x} \in A^*$.

**Theorem 23 (Implementation & Coherence).** A stream transducer $\mathcal{G} : \mathsf{G}(A, B)$ is coherent if and only if it implements some stream transduction.

*Proof.* Suppose that $\mathcal{G} = (\mathsf{St}, \mathsf{init}, \mathsf{o}, \mathsf{next}, \mathsf{out}) : \mathsf{G}(A, B)$ is a coherent transducer. Define the function $\beta : A \to B$ by $\beta(x) = \mathsf{o} \cdot \mathsf{out}(\mathsf{init}, x)$ for every $x \in A$, and the function $\mu : A \times A \to B$ by $\mu(x, y) = \mathsf{out}(\mathsf{next}(\mathsf{init}, x), y)$ for all $x, y \in A$. For any $x, y \in A$, we have to establish that $\beta(x) \cdot \mu(x, y) = \beta(xy)$. This follows immediately from Part (O2) of the coherence property for $\mathcal{G}$. So, $\langle \beta, \mu \rangle$ is a stream transduction. It remains to prove that $\mathcal{G}$ implements $\langle \beta, \mu \rangle$, that is,

$[\![\mathcal{G}]\!](\bar{x}) = \mathsf{F}(\beta,\mu)(\bar{x})$ for every $\bar{x} \in A^*$. For the base case, we have $[\![\mathcal{G}]\!](\varepsilon) = \langle \mathsf{o} \rangle$ and $\mathsf{F}(\beta,\mu)(\varepsilon) = \langle \beta(1) \rangle$, which are equal because $\beta(1) = \mathsf{o} \cdot \mathsf{out}(\mathsf{init}, 1) = \mathsf{o}$ by (O1). For the step case, we observe that:

$$[\![\mathcal{G}]\!](\bar{x} \cdot \langle y \rangle) = [\![\mathcal{G}]\!](\bar{x}) \cdot \langle \mathsf{out}(\mathsf{gnext}(\mathsf{init}, \bar{x}), y) \rangle \qquad \text{[Equation (4)]}$$
$$\mathsf{F}(\beta,\mu)(\bar{x} \cdot \langle y \rangle) = \mathsf{F}(\beta,\mu)(\bar{x}) \cdot \langle \mu(\pi(\bar{x}), y) \rangle \qquad \text{[def. of } \mathsf{F}(\beta,\mu)]$$

By the induction hypothesis, it suffices to show that $\mathsf{out}(\mathsf{gnext}(\mathsf{init}, \bar{x}), y)$ is equal to $\mu(\pi(\bar{x}), y) = \mathsf{out}(\mathsf{next}(\mathsf{init}, \pi(\bar{x})), y)$. This follows from the fact that $\mathsf{gnext}(\mathsf{init}, \bar{x})$ and $\mathsf{next}(\mathsf{init}, \pi(\bar{x}))$ are bisimilar, see Property (N*).

For the converse, suppose that $\mathcal{G} = (\mathsf{St}, \mathsf{init}, \mathsf{o}, \mathsf{next}, \mathsf{out}) : \mathsf{G}(A, B)$ is a transducer that implements $\langle \beta, \mu \rangle : \mathsf{STrans}(A, B)$. Define the relation $R$ as:

$$R = \{(s,t) \in \mathsf{St} \times \mathsf{St} \mid \text{there are } \bar{x}, \bar{y} \in A^* \text{ with } \pi(\bar{x}) = \pi(\bar{y}) \text{ s.t.}$$
$$s = \mathsf{gnext}(\mathsf{init}, \bar{x}) \text{ and } t = \mathsf{gnext}(\mathsf{init}, \bar{y})\}.$$

We claim that $R$ is a bisimulation. Consider arbitrary states $s, t \in \mathsf{St}$ with $sRt$ and $z \in A$. It follows that there are $\bar{x}, \bar{y} \in A^*$ with $\pi(\bar{x}) = \pi(\bar{y})$ such that $s = \mathsf{gnext}(\mathsf{init}, \bar{x})$ and $t = \mathsf{gnext}(\mathsf{init}, \bar{y})$. We have to show that $\mathsf{out}(s, z) = \mathsf{out}(t, z)$ and $\mathsf{next}(s, z)\, R\, \mathsf{next}(t, z)$. First, notice that:

$$[\![\mathcal{G}]\!](\bar{x} \cdot \langle z \rangle) = [\![\mathcal{G}]\!](\bar{x}) \cdot \langle \mathsf{out}(s, z) \rangle \qquad \text{[Equation (4), def. of } s]$$
$$\mathsf{F}(\beta,\mu)(\bar{x} \cdot \langle z \rangle) = \mathsf{F}(\beta,\mu)(\bar{x}) \cdot \langle \mu(\pi(\bar{x}), z) \rangle \qquad \text{[def. of } \mathsf{F}(\beta,\mu)]$$

Since $\mathcal{G}$ implements $\langle \beta, \mu \rangle$, we have that $[\![\mathcal{G}]\!](\bar{x} \cdot \langle z \rangle) = \mathsf{F}(\beta,\mu)(\bar{x} \cdot \langle z \rangle)$ and therefore $\mathsf{out}(s, z) = \mu(\pi(\bar{x}), z)$. Similarly, we can obtain that $\mathsf{out}(t, z) = \mu(\pi(\bar{y}), z)$. From $\pi(\bar{x}) = \pi(\bar{y})$ we get that $\mu(\pi(\bar{x}), z) = \mu(\pi(\bar{y}), z)$, and therefore $\mathsf{out}(s, z) = \mathsf{out}(t, z)$. Now, observe that $s' = \mathsf{next}(s, z) = \mathsf{next}(\mathsf{gnext}(\mathsf{init}, \bar{x}), z) = \mathsf{gnext}(\bar{x} \cdot \langle z \rangle)$ using Property 1. Similarly, we have that $t' = \mathsf{next}(t, z) = \mathsf{gnext}(\bar{y} \cdot \langle z \rangle)$. From $\pi(\bar{x} \cdot \langle z \rangle) = \pi(\bar{x})z = \pi(\bar{y})z = \pi(\bar{y} \cdot \langle z \rangle)$ we conclude that $s'Rt'$. We have thus established that $R$ is a bisimulation.

Now, we are ready to prove that $\mathcal{G}$ is coherent. We will only present the cases of Part (N2) and Part (O2), since they are the most interesting ones. Let $x, y \in A$. For Part (N2), we have to show that the states $s = \mathsf{next}(\mathsf{next}(\mathsf{init}, x), y)$ and $t = \mathsf{next}(\mathsf{init}, xy)$ are bisimilar. Since $R$ (previous paragraph) is a bisimulation, it suffices to show that $(s, t) \in R$. Indeed, this is true because $s = \mathsf{gnext}(\mathsf{init}, \langle x, y \rangle)$, $t = \mathsf{gnext}(\mathsf{init}, \langle xy \rangle)$ and $\pi(\langle x, y \rangle) = xy = \pi(\langle xy \rangle)$. For Part (O2), we have that $[\![G]\!](\langle xy \rangle) = \langle \mathsf{o}, \mathsf{out}(\mathsf{init}, xy) \rangle$ and $\mathsf{F}(\beta,\mu)(\langle xy \rangle) = \langle \beta(1), \mu(1, xy) \rangle$, as well as

$$[\![\mathcal{G}]\!](\langle x, y \rangle) = \langle \mathsf{o}, \mathsf{out}(\mathsf{init}, x), \mathsf{out}(\mathsf{next}(\mathsf{init}, x), y) \rangle \text{ and}$$
$$\mathsf{F}(\beta,\mu)(\langle x, y \rangle) = \langle \beta(1), \mu(1, x), \mu(x, y) \rangle,$$

using the definitions of $[\![G]\!]$ and $\mathsf{F}$. Since $\mathcal{G}$ implements $\langle \beta, \mu \rangle$, we know that $[\![\mathcal{G}]\!](\langle x, y \rangle) = \mathsf{F}(\beta,\mu)(\langle x, y \rangle)$ and $[\![G]\!](\langle xy \rangle) = \mathsf{F}(\beta,\mu)(\langle xy \rangle)$. Using all the above, we get that $\mathsf{o} \cdot \mathsf{out}(\mathsf{init}, x) \cdot \mathsf{out}(\mathsf{next}(\mathsf{init}, x), y) = \beta(1) \cdot \mu(1, x) \cdot \mu(x, y) = \beta(x) \cdot \mu(x, y) = \beta(xy)$ and $\mathsf{o} \cdot \mathsf{out}(\mathsf{init}, xy) = \beta(1) \cdot \mu(1, xy) = \beta(xy)$. So, Part (O2) of the coherence property holds. $\square$

Theorem 23 provides justification for our definition of the coherence property for stream transducers (recall Definition 20). It says that the definition is exactly appropriate, because it is a necessary and sufficient condition for a stream transducer to have a stream transduction as its denotation. In other words, the coherence property characterizes the transducers have a well-defined denotational semantics in terms of transductions. It offers this guarantee of correctness without limiting their expressive power as implementations of transductions.

**Theorem 24 (Expressive Completeness).** Let $A$ and $B$ be monoids, and $\langle\beta, \mu\rangle$ be a stream transduction in $\mathsf{STrans}(A, B)$. There exists a coherent stream transducer that implements $\langle\beta, \mu\rangle$.

*Proof.* Recall from Definition 8 that the monotonicity witness function $\mu$ satisfies the following property: $\beta(x) \cdot \mu(x, y) = \beta(xy)$ for every $x, y \in A$. Now, we define the transducer $\mathcal{G} = (\mathsf{St}, \mathsf{init}, \mathsf{o}, \mathsf{next}, \mathsf{out})$ as follows: $\mathsf{St} = A$, $\mathsf{init} = 1$, $\mathsf{o} = \beta(1)$, $\mathsf{next}(s, x) = s \cdot x$ and $\mathsf{out}(s, x) = \mu(s, x)$ for every state $s \in \mathsf{St}$ and input $x \in A$. The following properties hold for every $s \in \mathsf{St}$ and $\langle x_1, \ldots, x_n\rangle \in A^*$:

$$\mathsf{gnext}(s, \langle x_1, \ldots, x_n\rangle) = s \cdot x_1 \cdots x_n \quad \text{and} \tag{5}$$

$$\langle \mathsf{o}\rangle \cdot \mathsf{eout}(\mathsf{init}, \langle x_1, \ldots, x_n\rangle) = \mathsf{F}(\beta, \mu)(\langle x_1, \ldots, x_n\rangle) \tag{6}$$

Both these properties are shown by induction on the sequence $\langle x_1, \ldots, x_n\rangle$. It follows that $[\![G]\!](\bar{x}) = \langle \mathsf{o}\rangle \cdot \mathsf{eout}(\mathsf{init}, \bar{x}) = \mathsf{F}(\beta, \mu)(\bar{x})$ for every $\bar{x} \in A^*$. So, $\mathcal{G}$ implements the transduction $\langle\beta, \mu\rangle$. Finally, $\mathcal{G}$ is coherent by Theorem 23. □

Theorem 24 assures us that the abstract computational model of coherent stream transducers is expressive enough to implement any stream transduction. For this reason, we will be using stream transducers as the basic programming model for describing streaming computations.

**Example 25 (Correctness of Flatten).** Using induction, we will show that the transducer $\mathcal{G} = \mathtt{Flatten}(A) = (\mathsf{Unit}, \star, 1_A, \mathsf{next}, \mathsf{out})$ implements the transduction $\langle\pi, \mu\rangle = \mathit{flatten}(A)$ for a monoid $A$ (recall Examples 12 and 17). We show by induction that $[\![\mathcal{G}]\!](\bar{x}) = \mathsf{F}(\pi, \mu)(\bar{x})$ for every $\bar{x} \in \mathsf{FSeq}(A)^*$. For the base case, we have that $[\![\mathcal{G}]\!](\varepsilon) = \langle 1_A\rangle$ and $\mathsf{F}(\pi, \mu)(\varepsilon) = \langle\pi(\varepsilon)\rangle = \langle 1_A\rangle$. Now,

$$\begin{aligned}
[\![\mathcal{G}]\!](\bar{x} \cdot \langle y\rangle) &= [\![\mathcal{G}]\!](\bar{x}) \cdot \langle \mathsf{out}(\mathsf{gnext}(\mathsf{init}, \bar{x}), y)\rangle && [\text{def. of } [\![\mathcal{G}]\!]] \\
&= \mathsf{F}(\pi, \mu)(\bar{x}) \cdot \langle\pi(y)\rangle && [\text{I.H. and def. of } \mathsf{out}] \\
&= \mathsf{F}(\pi, \mu)(\bar{x}) \cdot \langle\mu(\pi(\bar{x}), y)\rangle && [\text{def. of } \mu] \\
&= \mathsf{F}(\pi, \mu)(\bar{x} \cdot \langle y\rangle) && [\text{def. of } \mathsf{F}]
\end{aligned}$$

for all $\bar{x} \in \mathsf{FSeq}(A)^*$ and $y \in \mathsf{FSeq}(A)$. We have thus proved that $\mathtt{Flatten}(A)$ is correct: its denotation is equal to the intended semantics.

**Example 26 (Correctness of Split).** We will establish that the transducer for splitting in batches is correct, namely that $\mathcal{G} = \mathtt{Split}(r) = (A, 1_A, \varepsilon, \mathsf{next}, \mathsf{out})$ implements $\langle r_1, \mu\rangle = \mathit{split}(r)$ for a splitter $r = (r_1, r_2)$ for the monoid $A$ (recall

Examples 13 and 18). Using the properties of splitters and an argument by induction, we obtain that $\mathsf{gnext}(\mathsf{init}, \bar{x}) = r_2(\pi(\bar{x}))$ for every $\bar{x} \in A^*$. We show by induction that $[\![\mathcal{G}]\!](\bar{x}) = \mathsf{F}(r_1, \mu)(\bar{x})$ for every $\bar{x} \in A^*$. For the base case, we have that $[\![\mathcal{G}]\!](\varepsilon) = \langle\varepsilon\rangle$ and $\mathsf{F}(r_1, \mu)(\varepsilon) = \langle r_1(1_A)\rangle = \langle\varepsilon\rangle$. Now,

$$\begin{aligned}
[\![\mathcal{G}]\!](\bar{x} \cdot \langle y\rangle) &= [\![\mathcal{G}]\!](\bar{x}) \cdot \langle \mathsf{out}(\mathsf{gnext}(\mathsf{init}, \bar{x}), y)\rangle && \text{[Equation (4)]} \\
&= \mathsf{F}(r_1, \mu)(\bar{x}) \cdot \langle \mathsf{out}(r_2(\pi(\bar{x})), y)\rangle && \text{[I.H. and previous claim]} \\
&= \mathsf{F}(r_1, \mu)(\bar{x}) \cdot \langle r_1(r_2(\pi(\bar{x})) \cdot y)\rangle && \text{[def. of } \mathsf{out}] \\
&= \mathsf{F}(r_1, \mu)(\bar{x}) \cdot \langle \mu(\pi(\bar{x}), y)\rangle && \text{[def. of } \mu] \\
&= \mathsf{F}(r_1, \mu)(\bar{x} \cdot \langle y\rangle) && \text{[def. of } \mathsf{F}]
\end{aligned}$$

for all $\bar{x} \in A^*$ and $y \in A$. We have thus established that `Split`$(r)$ is correct: its denotation is equal to the intended semantics.

## 5 Combinators for Deterministic Dataflow

We consider four dataflow combinators: (1) the *lifting* of pure morphisms to streaming computations, (2) *serial composition* for exposing pipeline parallelism, (3) *parallel composition* for exposing task-based parallelism, and (4) *feedback composition* for describing computations whose current output depends on previously produced output. The combinators are defined both for stream transductions (semantic objects) and for stream transducers (programs). Table 1 shows the definitions. The lifting of pure morphisms is implemented with a stateless transducer (i.e., the state space is a singleton set). Both parallel and serial composition are implemented using a product construction on transducers. In the case of parallel composition, each component computes independently. In the case of serial composition, the output of the first component is passed as input to the second component. In the case of feedback composition, the computation proceeds in well-defined rounds in order to prevent divergence.

We prove a precise correspondence between the semantics-level and program-level combinators for all cases: lifting (Proposition 27), parallel composition (Propsition 28), serial composition (Proposition 29), and feedback composition (Proposition 30). These are essentially ***correctness properties*** for the implementations of the combinators `Lift`, `Par`, `Serial`, `Loop`. They establish that our typed framework is appropriate for the modular specification of complex streaming computations, as it can support composition constructs that are essential for parallelization and distribution.

**Proposition 27 (Lifting).** Let $h : A \to B$ be a monoid homomorphism. Then, `Lift`$(h)$ is a coherent transducer and it implements the transduction $\mathrm{lift}(h)$.

**Proposition 28 (Parallel Composition).** Let $A_1$, $A_2$, $B_1$, $B_2$ be monoids, $\langle\beta_1, \mu_1\rangle : \mathsf{STrans}(A_1, B_1)$ and $\langle\beta_2, \mu_2\rangle : \mathsf{STrans}(A_2, B_2)$ be transductions, and $\mathcal{G}_1 : \mathsf{G}(A_1, B_1)$ and $\mathcal{G}_2 : \mathsf{G}(A_2, B_2)$ be transducers.

(1) Implementation: If $\mathcal{G}_1$ implements $\langle\beta_1, \mu_1\rangle$ and $\mathcal{G}_2$ implements $\langle\beta_2, \mu_2\rangle$, then `Par`$(\mathcal{G}_1, \mathcal{G}_2)$ implements $\langle\beta_1, \mu_1\rangle \parallel \langle\beta_2, \mu_2\rangle$.

**Table 1.** Combinators for deterministic dataflow.

***Lifting of monoid homomorphisms***

$$\frac{\text{monoid homomorphism } h : A \to B}{\mathrm{lift}(h) = \langle \beta, \mu \rangle : \mathsf{STrans}(A, B)} \qquad \begin{aligned} \beta(x) &= h(x) \\ \mu(x, y) &= h(y) \end{aligned}$$

$$\begin{aligned} \mathtt{Lift}(h) &= (\mathsf{St}, \mathsf{init}, \mathsf{o}, \mathsf{next}, \mathsf{out}) & \mathsf{init} &= \star & \mathsf{next}(s, x) &= s \\ \mathsf{St} &= \mathsf{Unit} & \mathsf{o} &= h(1) & \mathsf{out}(s, x) &= h(x) \end{aligned}$$

***Parallel composition***

$$\frac{\langle \beta_1, \mu_1 \rangle : \mathsf{STrans}(A_1, B_1) \qquad \langle \beta_2, \mu_2 \rangle : \mathsf{STrans}(A_2, B_2)}{\langle \beta_1, \mu_1 \rangle \parallel \langle \beta_2, \mu_2 \rangle = \langle \beta, \mu \rangle : \mathsf{STrans}(A_1 \times A_2, B_1 \times B_2)}$$

$$\beta(\langle x_1, x_2 \rangle) = \langle \beta_1(x_1), \beta_2(x_2) \rangle \qquad \mu(\langle x_1, x_2 \rangle, \langle y_1, y_2 \rangle) = \langle \mu_1(x_1, y_1), \mu_2(x_2, y_2) \rangle$$

$$\begin{aligned} \mathcal{G}_1 &= (\mathsf{St}_1, \mathsf{init}_1, \mathsf{o}_1, \mathsf{next}_1, \mathsf{out}_1) & \mathsf{init} &= \langle \mathsf{init}_1, \mathsf{init}_2 \rangle \\ \mathcal{G}_2 &= (\mathsf{St}_2, \mathsf{init}_2, \mathsf{o}_2, \mathsf{next}_2, \mathsf{out}_2) & \mathsf{o} &= \langle \mathsf{o}_1, \mathsf{o}_2 \rangle \\ \mathtt{Par}(\mathcal{G}_1, \mathcal{G}_2) &= (\mathsf{St}, \mathsf{init}, \mathsf{o}, \mathsf{next}, \mathsf{out}) & \mathsf{next}(\langle s_1, s_2 \rangle, \langle a, c \rangle) &= \langle \mathsf{next}_1(s_1, a), \mathsf{next}_2(s_2, c) \rangle \\ \mathsf{St} &= \mathsf{St}_1 \times \mathsf{St}_2 & \mathsf{out}(\langle s_1, s_2 \rangle, \langle a, c \rangle) &= \langle \mathsf{out}_1(s_1, a), \mathsf{out}_2(s_2, c) \rangle \end{aligned}$$

***Serial composition***

$$\frac{\langle \beta_1, \mu_1 \rangle : \mathsf{STrans}(A, B) \qquad \langle \beta_2, \mu_2 \rangle : \mathsf{STrans}(B, C)}{\langle \beta_1, \mu_1 \rangle \gg \langle \beta_2, \mu_2 \rangle = \langle \beta, \mu \rangle : \mathsf{STrans}(A, C)} \qquad \begin{aligned} \beta(x) &= \beta_2(\beta_1(x)) \\ \mu(x, y) &= \mu_2(\beta_1(x), \mu_1(x, y)) \end{aligned}$$

$$\begin{aligned} \mathcal{G}_1 &= (\mathsf{St}_1, \mathsf{init}_1, \mathsf{o}_1, \mathsf{next}_1, \mathsf{out}_1) & \mathsf{o} &= \mathsf{o}_2 \cdot \mathsf{out}_2(\mathsf{init}_2, \mathsf{o}_1) \\ \mathcal{G}_2 &= (\mathsf{St}_2, \mathsf{init}_2, \mathsf{o}_2, \mathsf{next}_2, \mathsf{out}_2) & \mathsf{next}(\langle s_1, s_2 \rangle, a) &= \langle \mathsf{next}_1(s_1, a), \\ \mathtt{Serial}(\mathcal{G}_1, \mathcal{G}_2) &= (\mathsf{St}_1 \times \mathsf{St}_2, \mathsf{init}, \mathsf{o}, \mathsf{next}, \mathsf{out}) & &\quad \mathsf{next}_2(s_2, \mathsf{out}_1(s_1, a)) \rangle \\ \mathsf{init} &= \langle \mathsf{init}_1, \mathsf{next}_2(\mathsf{init}_2, \mathsf{o}_1) \rangle & \mathsf{out}(\langle s_1, s_2 \rangle, a) &= \mathsf{out}_2(s_2, \mathsf{out}_1(s_1, a)) \end{aligned}$$

***Feedback composition***

$$\frac{\langle \beta, \mu \rangle : \mathsf{STrans}(A \times B, B)}{\mathit{loopB}(\beta, \mu) = \langle \gamma, \nu \rangle : \mathsf{STrans}(\mathsf{FSeq}(A), \mathsf{FSeq}(B))}$$

$$\begin{aligned} \gamma(\langle a_1, \ldots, a_n \rangle) &= \langle b_0, b_1, \ldots, b_n \rangle \\ \gamma(\varepsilon) &= \langle b_0 \rangle, \text{ where } b_0 = \beta(1_A, 1_B) \\ \gamma(\langle a_1, \ldots, a_n, a_{n+1} \rangle) &= \gamma(\langle a_1, \ldots, a_n \rangle) \cdot \langle b_{n+1} \rangle, \text{ where} \\ b_{n+1} &= \mu(\langle a_1 \cdots a_n, b_0 b_1 \cdots b_{n-1} \rangle, \langle a_{n+1}, b_n \rangle) \end{aligned}$$

$$\begin{aligned} \mathcal{G} &= (\mathsf{St}, \mathsf{init}, \mathsf{o}, \mathsf{next}, \mathsf{out}) : \mathsf{G}(A \times B, B) \\ \mathtt{LoopB}(\mathcal{G}) &= (\mathsf{St}', \mathsf{init}', \mathsf{o}', \mathsf{next}', \mathsf{out}') : \mathsf{G}(\mathsf{FSeq}(A), \mathsf{FSeq}(B)) \\ \mathsf{St}' &= \mathsf{St} \times B \quad \text{(second component: last output batch)} \\ \mathsf{init}' &= \langle \mathsf{init}, \mathsf{o} \rangle \text{ and } \mathsf{o}' = \langle \mathsf{o} \rangle \\ \mathsf{next}'(\langle s, b \rangle, a) &= \langle \mathsf{next}(s, \langle a, b \rangle), \mathsf{out}(s, \langle a, b \rangle) \rangle \\ \mathsf{out}'(\langle s, b \rangle, a) &= \langle \mathsf{out}(s, \langle a, b \rangle) \rangle \end{aligned}$$

$$\frac{\langle \beta, \mu \rangle : \mathsf{STrans}(A \times B, B) \qquad \text{splitter } r \text{ for } A}{\mathit{loop}(\beta, \mu, r) = \mathit{split}(r) \gg \mathit{loopB}(\beta, \mu) \gg \mathit{flatten}(B) : \mathsf{STrans}(A, B)}$$

$$\frac{\mathcal{G} : \mathsf{G}(A \times B, B) \qquad \text{splitter } r \text{ for } A}{\mathtt{Loop}(\mathcal{G}, r) = \mathtt{Serial}(\mathtt{Split}(r), \mathtt{LoopB}(\mathcal{G}), \mathtt{Flatten}(B)) : \mathsf{G}(A, B)}$$

(2) Coherence: If $\mathcal{G}_1$ and $\mathcal{G}_2$ are coherent, then so is $\texttt{Par}(\mathcal{G}_1, \mathcal{G}_2)$.

*Proof.* Notice that Part (2) follows immediately from Part (1) and Theorem 23. Define $f = [\![\texttt{Par}(\mathcal{G}_1, \mathcal{G}_2)]\!]$ and $\langle\beta, \mu\rangle = \langle\beta_1, \mu_1\rangle \parallel \langle\beta_2, \mu_2\rangle$. We will show that $f(\bar{w}) = \mathsf{F}(\beta, \mu)(\bar{w})$ for every $\bar{w} \in (A_1 \times A_2)^*$. Suppose that $\mathsf{fst}$ is the (elementwise) left projection function. We claim that $\mathsf{fst}(\mathsf{gnext}(s, \bar{w})) = \mathsf{gnext}_1(\mathsf{fst}(s), \mathsf{fst}(\bar{w}))$ and $\mathsf{fst}(\mathsf{eout}(s, \bar{w})) = \mathsf{eout}_1(\mathsf{fst}(s), \mathsf{fst}(\bar{w}))$ for all $s \in \mathsf{St}$ and $\bar{w} \in (A_1 \times A_2)^*$. Both claims are shown by induction on the length of $\bar{w}$. With similar arguments we can obtain that $\mathsf{snd}(f(\bar{w})) = [\![\mathcal{G}_2]\!](\mathsf{snd}(\bar{w}))$ for every $\bar{w} \in (A_1 \times A_2)^*$. It can be shown by induction that $\mathsf{fst}(\mathsf{F}(\beta, \mu)(\bar{w})) = \mathsf{F}(\beta_1, \mu_1)(\mathsf{fst}(\bar{w}))$ and $\mathsf{snd}(\mathsf{F}(\beta, \mu)(\bar{w})) = \mathsf{F}(\beta_1, \mu_1)(\mathsf{snd}(\bar{w}))$ for all $\bar{w} \in (A_1 \times A_2)^*$. In order to establish that $f(\bar{w}) = \mathsf{F}(\beta, \mu)(\bar{w})$, it suffices to show that $\mathsf{fst}(f(\bar{w})) = \mathsf{fst}(\mathsf{F}(\beta, \mu)(\bar{w}))$ and $\mathsf{snd}(f(\bar{w})) = \mathsf{snd}(\mathsf{F}(\beta, \mu)(\bar{w}))$. Given the claims shown previously, these equalities are equivalent to $[\![\mathcal{G}_1]\!](\mathsf{fst}(\bar{w})) = \mathsf{F}(\beta_1, \mu_1)(\mathsf{fst}(\bar{w}))$ and $[\![\mathcal{G}_2]\!](\mathsf{snd}(\bar{w})) = \mathsf{F}(\beta_2, \mu_2)(\mathsf{snd}(\bar{w}))$ respectively. These equalities follow from the assumptions that $\mathcal{G}_1$ implements $\langle\beta_1, \mu_1\rangle$ and $\mathcal{G}_2$ implements $\langle\beta_2, \mu_2\rangle$. □

**Proposition 29 (Serial Composition).** Let $A$, $B$, $C$ be monoids, $\langle\beta_1, \mu_1\rangle : \mathsf{STrans}(A, B)$ and $\langle\beta_2, \mu_2\rangle : \mathsf{STrans}(B, C)$ be transductions, and $\mathcal{G}_1 : \mathsf{G}(A, B)$ and $\mathcal{G}_2 : \mathsf{G}(B, C)$ be transducers.

(1) Implementation: If $\mathcal{G}_1$ implements $\langle\beta_1, \mu_1\rangle$ and $\mathcal{G}_2$ implements $\langle\beta_2, \mu_2\rangle$, then $\texttt{Serial}(\mathcal{G}_1, \mathcal{G}_2)$ implements $\langle\beta_1, \mu_1\rangle \gg \langle\beta_2, \mu_2\rangle$.

(2) Coherence: If $\mathcal{G}_1$ and $\mathcal{G}_2$ are coherent, then so is $\texttt{Serial}(\mathcal{G}_1, \mathcal{G}_2)$.

*Proof.* Part (2) follows easily from Part (1) and Theorem 23. In order to prove Part (1) we have to first establish a number of preliminary facts. We define the function $\mathsf{M}_2 : A^* \to A$ as follows: $\mathsf{M}_2(\varepsilon) = 1$, $\mathsf{M}_2(\langle x\rangle) = x$ for $x \in A$, and $\mathsf{M}_2(\langle x, y\rangle \cdot \bar{z}) = \langle xy\rangle \cdot \bar{z}$ for $x, y \in A$ and $\bar{z} \in A^*$. We write $\mathcal{G}$ to denote $\mathcal{G}_1 \gg \mathcal{G}_2$.

$$\mathsf{fst}(\mathsf{gnext}(s, \bar{x})) = \mathsf{gnext}_1(\mathsf{fst}(s), \bar{x}) \text{ for all } s \in \mathsf{St} \text{ and } \bar{x} \in A^* \tag{7}$$

$$\mathsf{snd}(\mathsf{gnext}(s, \bar{x})) = \mathsf{gnext}_2(\mathsf{snd}(s), \mathsf{eout}_1(\mathsf{fst}(s), \bar{x})) \text{ for all } s \in \mathsf{St} \text{ and } \bar{x} \in A^* \tag{8}$$

$$[\![\mathcal{G}]\!](\bar{x}) = \mathsf{M}_2([\![\mathcal{G}_2]\!]([\![\mathcal{G}_1]\!](\bar{x}))) \text{ for all } \bar{x} \in A^* \tag{9}$$

$$\mathsf{F}(\beta, \mu)(\bar{x}) = \mathsf{M}_2(\mathsf{F}(\beta_2, \mu_2)(\mathsf{F}(\beta_1, \mu_1)(\bar{x}))) \text{ for all } \bar{x} \in A^* \tag{10}$$

where $\langle\beta, \mu\rangle = \langle\beta_1, \mu_1\rangle \gg \langle\beta_2, \mu_2\rangle$. All four claims above are proved by induction on the sequence $\bar{x}$. Equations (7) and (8) are needed to prove Equation (9). Now, we will establish that $\mathcal{G}$ implements $\langle\beta, \mu\rangle$. Indeed, we have that

$$\begin{aligned}
[\![\mathcal{G}]\!](\bar{x}) &= \mathsf{M}_2([\![\mathcal{G}_2]\!]([\![\mathcal{G}_1]\!](\bar{x}))) && \text{[Equation (9)]} \\
&= \mathsf{M}_2([\![\mathcal{G}_2]\!](\mathsf{F}(\beta_1, \mu_1)(\bar{x}))) && [\mathcal{G}_1 \text{ implements } \langle\beta_1, \mu_1\rangle] \\
&= \mathsf{M}_2(\mathsf{F}(\beta_2, \mu_2)(\mathsf{F}(\beta_1, \mu_1)(\bar{x}))) && [\mathcal{G}_2 \text{ implements } \langle\beta_2, \mu_2\rangle] \\
&= \mathsf{F}(\beta, \mu)(\bar{x}) && \text{[Equation (10)]}
\end{aligned}$$

for every $\bar{x} \in A^*$. So, we conclude that $\mathcal{G}$ implements $\langle\beta, \mu\rangle$. □

Let us give an example of how to construct complex computations from simpler ones using the dataflow combinators. Let $A, B$ be sets and $\mathsf{op} : A \to B$

be a function. We want to describe a streaming computation with two input channels, both of type $\mathsf{FBag}(A)$, and one output channel of type $\mathsf{FBag}(B)$. The computation transforms both input channels in the same way, namely by applying the function $\mathsf{op}$ to each element. This gives two output substreams, both of type $\mathsf{FBag}(B)$, that are merged into the output stream. The function $\mathsf{op} : A \to B$ lifts to a monoid homomorphism $\mathsf{op} : \mathsf{FBag}(A) \to \mathsf{FBag}(B)$, given by $\mathsf{op}(x) = \{\mathsf{op}(a) \mid a \in x\}$ for every multiset $x$. The streaming computation described previously can be visualized using the dataflow graph shown below.

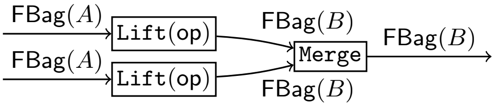

Each edge of the graph represents a communication channel along which a stream flows, and it is annotated with the type of the stream. The dataflow graph above represents the transducer $\mathcal{G} = \mathtt{Serial}(\mathtt{Par}(\mathtt{Lift}(\mathsf{op}), \mathtt{Lift}(\mathsf{op})), \mathtt{Merge})$, where $\mathtt{Merge} : \mathsf{G}(\mathsf{FBag}(A) \times \mathsf{FBag}(A), \mathsf{FBag}(A))$ is the transducer of Example 16. From Propositions 27, 29 and 28 we obtain that $\mathcal{G}$ implements the transduction $(\mathrm{lift}(\mathsf{op}) \parallel \mathrm{lift}(\mathsf{op})) \gg \mathrm{merge}$, where merge is described in Example 11.

We will now consider the feedback combinator, which introduces cycles in the dataflow graph. One consequence of cyclic graphs in the style of Kahn-MacQueen [60] is that divergence can be introduced, that is, a finite amount of input can cause an operator to enter an infinite loop. For example, consider the transducer $\mathtt{Merge} : \mathsf{G}(\mathsf{FBag}(A) \times \mathsf{FBag}(A), \mathsf{FBag}(A))$ of Example 16. The figure below visualizes the dataflow graph, where the output channel of Merge is connected to one of its input channels, thus forming a feedback loop.

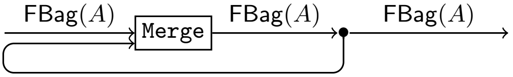

Suppose that the singleton input $\{a\}$ is fed to the input of the dataflow graph above, which corresponds to the first input channel of Merge. This will cause Merge to emit $\{a\}$, which will be sent again to the second input channel of Merge. Intuitively, this will cause the computation to enter an infinite loop (divergence) of consuming and emitting $\{a\}$. This behavior is undesirable in systems that process data streams, because divergence can make the system unresponsive. For this reason, we will consider here a form of feedback that eliminates this problem by ensuring that the computation of a feedback loop proceeds in a *sequence of rounds*. This avoid divergence, because the computation always makes progress by moving from one round to the next, as dictated by the input data. We describe this organization in rounds by requiring that the programmer specifies a *splitter* (recall Example 18). The splitter decomposes the input stream into *batches*, and one round of computation for the feedback loop corresponds to consuming one batch of data, generating the corresponding output batch, and sending the output batch along the feedback loop to be available for the next round of processing. This form of feedback allows flexibility in specifying what constitutes a single *batch* (and thus a single *round*), and therefore generalizes the feedback combinator of Synchronous Languages such as Lustre [31].

**Proposition 30 (Feedback Composition).** Let $A$ and $B$ be monoids, $\langle\beta,\mu\rangle$ : $\mathsf{STrans}(A,B)$ be a transduction, $\mathcal{G}$ : $\mathsf{G}(A,B)$ be a transducer, and $r = (r_1, r_2)$ be a splitter for $A$ (see Example 13).
(1) IMPLEM.: If $\mathcal{G}$ implements $\langle\beta,\mu\rangle$, then $\mathtt{Loop}(\mathcal{G},r)$ implements $loop(\beta,\mu,r)$.
(2) COHERENCE: If $\mathcal{G}$ is coherent, then so is $\mathtt{Loop}(\mathcal{G},r)$.

*Proof.* We leave to the reader the proofs that `Split` (Example 18) implements *split* and that `Flatten` (Example 17) implements *flatten*. Given Proposition 29, it suffices to show that $\mathcal{G}' = \mathtt{LoopB}(\mathcal{G})$ implements $\langle\gamma,\nu\rangle = loopB(\beta,\mu)$. Since $\mathcal{G}'$ is of type $\mathsf{G}(\mathsf{FSeq}(A),\mathsf{FSeq}(B))$ it suffices to define the transition and output functions on singleton sequences (as done in Table 1), because there is a unique way to extend them so that $\mathcal{G}'$ is coherent. It remains to show that $[\![\mathcal{G}']\!](\bar{x}) = \mathsf{F}(\gamma,\nu)(\bar{x})$ for every $\bar{x} \in \mathsf{FSeq}(A)^*$. The base case is easy, and for the step case it suffices to show that $\mathsf{out}'(\mathsf{gnext}'(\mathsf{init}',\bar{x}),y) = \nu(\pi(\bar{x}),y)$ for every $\bar{x} \in \mathsf{FSeq}(A)^*$ and $y \in \mathsf{FSeq}(A)$. As we discussed before, $\mathsf{gnext}'$ and $\mathsf{out}'$ can be viewed as being defined on elements of $A$ rather than sequences of $\mathsf{FSeq}(A)$, so we can equivalently prove that $\mathsf{out}'(\mathsf{gnext}'(\mathsf{init}',\langle a_1,\ldots,a_n\rangle),a_{n+1}) = \nu(\langle a_1,\ldots,a_n\rangle,a_{n+1})$ with each $a_i$ an element of $A$. Given that $\mathcal{G}$ implements $\langle\beta,\mu\rangle$, the key observation to finish the proof is $\mathsf{gnext}'(\mathsf{init}',\langle a_1,\ldots,a_n\rangle) = \langle \mathsf{gnext}(\mathsf{init},\langle\langle a_1,b_0\rangle,\ldots,\langle a_n,b_{n-1}\rangle\rangle),b_n\rangle$, where $\gamma(\langle a_1,\ldots,a_n\rangle) = \langle b_0,b_1,\ldots,b_n\rangle$. □

**Example 31.** For an example of using the feedback combinator, consider the transduction $\langle\beta,\mu\rangle$ which adds two input streams of numbers pointwise. That is, $\beta : \mathsf{FSeq}(\mathbb{N}) \times \mathsf{FSeq}(\mathbb{N}) \to \mathsf{FSeq}(\mathbb{N})$ is defined by $\beta(x_1x_2\ldots x_m, y_1y_2\ldots y_n) = 0(x_1+y_1)(x_2+y_2)\ldots(x_k+y_k)$ where $k = \min(m,n)$. Additionally, consider the trivial splitter $r = (r_1,r_2)$ for sequences where each batch is a singleton: $r_1(x_1\ldots x_n) = \langle x_1,\ldots,x_n\rangle$ and $r_2(x_1\ldots x_n) = \varepsilon$. We use this splitter to enforce that each batch is a single element and that each round of the computation involves consuming one element. Finally, the transduction $loop(\beta,\mu,r) = \langle\gamma,\nu\rangle$ describes the *running sum*, that is, $\gamma(x_1\ldots x_n) = 0x_1(x_1+x_2)\ldots(x_1+\cdots+x_n)$.

The dataflow combinators of this section could form the basis of query language design. The StreamQRE language [10,84] and related formalisms [9,11,12, 14] are based on a set of combinators for efficiently processing linearly-ordered streams (e.g., time series [3,4]). Extending a language like StreamQRE to the typed setting of stream transductions is an interesting research direction.

## 6 Algebraic Reasoning for Optimizing Transformations

Our typed denotational framework can be used to validate optimizing transformations using algebraic reasoning. This amounts to establishing that the original transducer is equivalent to the optimized one. A fundamental approach for showing equivalence of composite transducers is to establish algebraic laws between basic building blocks, and then use algebraic rewriting.

As a concrete example, consider the per-key streaming aggregation of Example 10, which is described by the transduction $\text{reduce}(K, \mathsf{op}) : \mathsf{STrans}(\mathsf{FBag}(K \times V), \mathsf{FMap}(K, V))$, where $K$ is the set of keys, $V$ is the set of values, and $\mathsf{op} : V \times V \to V$ is an associative and commutative aggregation operation. Let $h : K \to \{1, \ldots, n\}$ be a hash function for the keys, and define $K_i^h = h^{-1}(i) = \{k \in K \mid h(k) = i\}$ for every $i$. Consider two variants of the merging operation of Example 11: (1) $\text{kmerge}(h)$ merges $n$ input streams of types $\mathsf{FBag}(K_1^h \times V), \ldots,$ $\mathsf{FBag}(K_n^h \times V)$ respectively into an output stream of type $\mathsf{FBag}(K \times V)$, and (2) $\text{mmerge}(h)$ merges $n$ input streams of types $\mathsf{FMap}(K_1^h, V), \ldots, \mathsf{FMap}(K_n^h, V)$ respectively into an output stream of type $\mathsf{FMap}(K, V)$. We also consider the transduction $\text{ksplit}(h)$ that partitions an input stream of type $\mathsf{FBag}(K \times V)$ into $n$ output substreams of types $\mathsf{FBag}(K_1^h \times V), \ldots, \mathsf{FBag}(K_n^h \times V)$ respectively. Using elementary set-theoretic arguments, the following equalities can be established: $\text{ksplit}(h) \gg \text{kmerge}(h) = id$ and

$$\text{kmerge}(h) \gg \text{rd}(K, \mathsf{op}) = (\text{rd}(K_1^h, \mathsf{op}) \parallel \cdots \parallel \text{rd}(K_n^h, \mathsf{op})) \gg \text{mmerge}(h),$$

where rd abbreviates reduce. Next, we consider the corresponding transducers $\mathtt{KSplit}(h)$, $\mathtt{KMerge}(h)$, $\mathsf{Id}$, $\mathtt{Reduce}(K, \mathsf{op})$ (abbreviation $\mathtt{Rd}$) and $\mathtt{MMerge}(h)$ and establish that they implement the respective transductions. This can be shown with induction proofs as shown earlier in Example 25 and Example 26. Using these facts and the propositions of Sect. 5, the equalities between transductions shown earlier give the following equations (equivalences) between transducers: $\mathtt{KSplit}(h) \gg \mathtt{KMerge}(h) \equiv \mathsf{Id}$ and

$$\mathtt{KMerge}(h) \gg \mathtt{Rd}(K, \mathsf{op}) \equiv (\mathtt{Rd}(K_1^h, \mathsf{op}) \parallel \cdots \parallel \mathtt{Rd}(K_n^h, \mathsf{op})) \gg \mathtt{MMerge}(h).$$

Using these equations, we can establish the following optimizing transformation for *data parallelization*, which is useful when processing high-rate data streams.

$$\begin{aligned} \mathtt{Reduce}(K, \mathsf{op}) &\equiv \mathsf{Id} \gg \mathtt{Reduce}(K, \mathsf{op}) \\ &\equiv \mathtt{KSplit}(h) \gg \mathtt{KMerge}(h) \gg \mathtt{Reduce}(K, \mathsf{op}) \\ &\equiv \mathtt{KSplit}(h) \gg (\mathtt{Rd}(K_1^h, \mathsf{op}) \parallel \cdots \parallel \mathtt{Rd}(K_n^h, \mathsf{op})) \gg \mathtt{MMerge}(h). \end{aligned}$$

The above equation illustrates our proposed style of reasoning for establishing the soundness of optimizing streaming transformations: (1) prove equalities between transductions using elementary set-theoretic arguments, (2) prove that the transducers (programs) implement the transductions (denotations) using induction, (3) translate the equalities between transductions into equivalences between transducers using the results of Sect. 5, and finally (4) use algebraic reasoning to establish more complex equivalences.

The example of this section is simple but illustrates two key points: (1) our data types for streams (monoids) capture important invariants about the streams that enable transformations, and (2) useful program transformations can be established with denotational arguments that require an appropriate notion of transduction. This approach opens up the possibility of formally verifying the wealth of optimizing transformations that are used in stream processing systems.

The papers [54, 101] describe several of them, but use informal arguments that rely on the operational intuition about streaming computations. Our approach here, on the other hand, relies on rigorous denotational arguments.

The equational axiomatizations of arrows [56] and traced monoidal categories [58] are relevant to our setting, but would require adaptation. An interesting question is whether a *complete axiomatization* can be provided for the basic dataflow combinators of Sect. 5, similarly to how Kleene Algebra (KA) [62, 63] and its extensions [49,64,79,83] (as well as other program logics [65,66,78,80–82]) capture properties of imperative programs at the propositional level. We also leave for future work the development of the coalgebraic approach [96–98] for reasoning about the equivalence of stream transducers. We have already defined a notion of bisimulation in Sect. 4, which could give an alternative approach for proving equivalence using coinduction on the transducers.

## 7 Related Work

Sect. 1 contains several pointers to related literature for stream processing. In this section, we will focus on prior work that specifically addresses aspects of formal semantics for streaming computation.

The seminal work of Gilles Kahn [59] is exemplary in its rigorous treatment of denotational semantics for a language of ***deterministic dataflow*** graphs of independent processes, which access their input channels using blocking read statements and the output channels using nonblocking write statements. The language Lustre [31] is a synchronous restriction of Kahn's model, which introduces the semantic idea of a clock for specifying the rate of a stream. Other notable synchronous formalisms are the language Signal [21, 72] and Esterel [22, 28], and the synchronous dataflow graphs of [73] and [24]. These formalisms are all deterministic, in the sense the the output is determined purely by the input data. Nondeterminism creates unavoidable semantic complications [30].

The CQL language [16] is a streaming extension of a ***relational*** database language with additional constructs for time-based windowing. The denotational semantics of CQL [17] can be reconstructed and greatly simplified within our framework using the notion of stream described in Example 7 (finite time-varying multisets). There are several works that deal with the semantics of specific language constructs (e.g., windows), notions of time, punctuations and disordered streams, but do not give a mathematical description of the overall streaming computation [5, 7, 25, 44, 67, 75, 76, 109].

The literature on Functional Reactive Programming (***FRP***) [34, 46, 47, 55, 68, 69, 93, 103] is closely related to the deterministic dataflow formalisms mentioned earlier. The main abstractions in FRP are signals and event sequences, which are linearly ordered data. Processing unordered data (e.g., multisets and maps) and extracting data parallelism (e.g., the per-key aggregation of Sect. 6) require a data model that goes beyond linear orders. In particular, the axioms of *arrows* [56] (often used in FRP) cannot prove the soundness of the optimizing transformation of Sect. 6, which requires reasoning about multisets.

The idea of using ***types*** to classify streams has been recently explored in [85] (see also [13]), but only for a restricted class of types that correspond to partial orders. No general abstract model of computation is presented in [85], and many of the examples in this paper cannot be adequately accomodated.

The mathematical framework of ***coalgebras*** [97] has been used to describe streams [98]. One advantage of this approach is that proofs of equivalence can be given using the proof principle of coinduction [96], which in many cases offers a useful alternative to proofs by induction. This line of work mostly focuses on infinite sequences of elements, whereas here we focus on the transformation of streams of data that can be of various different forms (not just sequences).

The idea to model the input/output of automata using monoids has appeared in the ***algebraic theory*** of automata and transducers. Monoids (non-free, e.g. $A^* \times B^*$) have been used to generalize automata from recognizers of languages to recognizers of relations [45], which are sometimes called *rational transducers* [100]. Our focus here is on (deterministic) functions, as models that recognize relations can give rise to the Brock-Ackerman anomaly [30]. The automata models (with inputs from a free monoid $A^*$) most closely related to our stream transducers are deterministic: Mealy machines [87], Moore machines [90], sequential transducers [48, 95], and sub-sequential transducers [102]. The concept of coherence that we introduce here (Definition 20) does not arise in these models, because they do not operate on input batches. An algebraic generalization of a deterministic acceptor is provided by a *right monoid action* $\delta : \mathsf{St} \times A \to \mathsf{St}$ (see page 231 of [100]), which satisfies the following properties for all $s \in \mathsf{St}$ and $x, y \in A$: (1) $\delta(s, 1) = s$, and (2) $\delta(\delta(s, x), y) = \delta(s, xy)$. These properties look similar to (N1) and (N2) of Definition 20. They are, however, too restrictive for our stream transducers, as they would falsify Theorem 23.

## 8 Conclusion

We have presented a typed semantic framework for stream processing, based on the idea of abstracting data streams as elements of algebraic structures called *monoids*. Data streams are thus classified using monoids as *types*. Stream transformations are modeled as monotone functions, which are organized by input/output type. We have adapted the classical model of string transducers to our setting, and we have developed a general theory of streaming computation with a formal denotational semantics. The entire technical development in this paper is constructive, and therefore lends itself well to formalization in a proof assistant such as Coq [23, 35, 106]. Our framework can be used for the formalization of streaming models, and the validation of subtle optimizations of streaming programs (e.g., Sect. 6), such as the ones described in [54, 101]. We have restricted our attention in this paper to *deterministic* streaming computation, in the sense that the behaviors that we model have predictable and reproducible results. Nondeterminism causes fundamental semantic difficulties [30], and it is undesirable in applications where repeatability is important.
